# Deubiquitylating enzyme USP9x regulates radiosensitivity in glioblastoma cells by Mcl-1-dependent and -independent mechanisms

**DOI:** 10.1038/cddis.2015.405

**Published:** 2016-01-14

**Authors:** F Wolfsperger, S A Hogh-Binder, J Schittenhelm, T Psaras, V Ritter, L Bornes, S M Huber, V Jendrossek, J Rudner

**Affiliations:** 1 Department of Radiation Oncology, University of Tuebingen, Tuebingen, Germany; 2 Department of Neuropathology, Institute of Pathology and Neuropathology, University of Tuebingen, Tuebingen, Germany; 3 Department of Neurosurgery, University of Tuebingen, Tuebingen, Germany; 4 Institute for Cell Biology, University Hospital Essen, Essen, Germany

## Abstract

Glioblastoma is a very aggressive form of brain tumor with limited therapeutic options. Usually, glioblastoma is treated with ionizing radiation (IR) and chemotherapy after surgical removal. However, radiotherapy is frequently unsuccessful, among others owing to resistance mechanisms the tumor cells have developed. Antiapoptotic B-cell leukemia (Bcl)-2 family members can contribute to radioresistance by interfering with apoptosis induction in response to IR. Bcl-2 and the closely related Bcl-xL and Mcl-1 are often overexpressed in glioblastoma cells. In contrast to Bcl-2 and Bcl-xL, Mcl-1 is a short-lived protein whose stability is closely regulated by ubiquitylation-dependent proteasomal degradation. Although ubiquitin ligases facilitate degradation, the deubiquitylating enzyme ubiquitin-specific protease 9x (USP9x) interferes with degradation by removing polyubiquitin chains from Mcl-1, thereby stabilizing this protein. Thus, an inability to downregulate Mcl-1 by enhanced USP9x activity might contribute to radioresistance. Here we analyzed the impact of USP9x on Mcl-1 levels and radiosensitivity in glioblastoma cells. Correlating Mcl-1 and USP9x expressions were significantly higher in human glioblastoma than in astrocytoma. Downregulation of Mcl-1 correlated with apoptosis induction in established glioblastoma cell lines. Although Mcl-1 knockdown by siRNA increased apoptosis induction after irradiation in all glioblastoma cell lines, USP9x knockdown significantly improved radiation-induced apoptosis in one of four cell lines and slightly increased apoptosis in another cell line. In the latter two cell lines, USP9x knockdown also increased radiation-induced clonogenic death. The massive downregulation of Mcl-1 and apoptosis induction in A172 cells transfected with USP9x siRNA shows that the deubiquitinase regulates cell survival by regulating Mcl-1 levels. In contrast, USP9x regulated radiosensitivity in Ln229 cells without affecting Mcl-1 levels. We conclude that USP9x can control survival and radiosensitivity in glioblastoma cells by Mcl-1-dependent and Mcl-1-independent mechanisms.

Along with surgery, radiotherapy, and chemotherapy are the main treatment options of tumors. While the former aims to remove the tumor bulk mass, the latter two intend to neutralize remaining tumor cells. Ionizing radiation (IR) exerts its cytotoxic effects by inducing cell death. One form of specific cell death induced by IR is intrinsic apoptosis, which is regulated by members of the B-cell leukemia (Bcl)-2 protein family.^1^

The Bcl-2 protein family consists of protective antiapoptotic and pro-apoptotic members, which keep each other in check by antagonizing each other's function.^2^ The activation of pro-apoptotic multidomain proteins Bax and Bak is essential to induce mitochondrial outer membrane permeabilization, resulting in the release of cytochrome C and other apoptotic factors into the cytosol where, in turn, caspases become activated. Antiapoptotic Bcl-2 family members prevent the activation of Bax and Bak either by direct interaction or indirectly by sequestering pro-apoptotic BH3-only proteins Bim and Bid that are required to activate Bax and Bak. Other BH3-only proteins are also able to bind to antiapoptotic proteins, thereby releasing Bax and Bak from their inhibitory complexes with antiapoptotic proteins. Changing the balance between anti- and pro-apoptotic Bcl-2 family members can shift the cells toward survival or apoptosis, depending on whether the protective or the detrimental proteins dominate.

Bcl-2 itself, Bcl-xL, and myeloid cell lymphoma-1 (Mcl-1) belong to the antiapoptotic proteins of the Bcl-2 family. They are often overexpressed in tumor cells and are associated with increased resistance to apoptosis induction in response to radio- and chemotherapy.^3, 4^ As more than one of the protective proteins can be upregulated in tumors, the neutralization of all antiapoptotic proteins is needed to successfully induce apoptosis. Blocking the antiapoptotic function of Bcl-2/Bcl-xL by inhibitors mimicking BH3-only proteins, such as ABT737 and ABT263, can induce apoptosis in cells with low Mcl-1 levels but has no effect on cells with high Mcl-1 levels.^5, 6, 7^ In contrast, specific inhibitors targeting Mcl-1 have been insufficiently described until now. However, Mcl-1 availability might be modulated by targeting pathways that regulate Mcl-1 stability.

In contrast to Bcl-2 and Bcl-xL, Mcl-1 is a relatively short-lived protein.^8, 9^ Usually, Mcl-1 is quickly ubiquitylated by specific ubiquitin ligases and targeted for proteasomal degradation. Phosphorylation of Mcl-1, for example by glycogen synthase kinase GSK-3*β*, can accelerate this degrading process,^10, 11^ whereas deubiquitinases counteract it by removing the polyubiquitin chain, thereby stabilizing the short-lived protein. The ubiquitin-specific protease 9x (USP9x) was recently identified as a Mcl-1 specific deubiquitinase.^12^ However, the circumstances under which USP9x regulates Mcl-1 stability are not well understood. Schwickart *et al.*^12^ showed that USP9x levels correlated with Mcl-1 levels, suggesting a constitutive regulation of Mcl-1 levels by the deubiquitinase. In contrast, our recent results showed no effect of USP9x on Mcl-1 levels in healthy Jurkat cells, but an accelerated IR-induced Mcl-1 degradation was detected when USP9x was knocked down.^9^ This indicates that the association of USP9x with Mcl-1 is regulated by a yet unknown mechanism in response to irradiation.

In the present study, we aimed to analyze the impact of USP9x on Mcl-1 and cell survival in glioblastoma cell lines. Glioblastoma is not only the most common but also a very aggressive form of brain tumor that are primarily removed by surgery as radically as possible and consecutively treated with radiochemotherapy, if the patient's condition allows for adjuvant therapy.^13^ Despite the multimodal treatment, the median patient survival is below 1.5 years. Comparing human grade III astrocytoma with grade IV glioblastoma samples, we could show that Mcl-1 and USP9x are upregulated during tumor progression. Furthermore, we examined four established (A172, U373, Ln229, T98G) and two primary (LKI, WKI) glioblastoma cell lines that differ in their ability to downregulate Mcl-1 and induce apoptosis in response to IR. Analyzing A172 and U373 cells more closely, we detected an increased Mcl-1 ubiquitylation that correlated with a reduced Mcl-1 stability 48 h after irradiation in U373 cells, but not in A172 cells. Moreover, Mcl-1 knockdown sensitized A172, Ln229, and T98G cells to IR-induced apoptosis, suggesting that Mcl-1 is an important factor increasing glioblastoma cell survival after irradiation. In contrast, USP9x knockdown slightly increased apoptosis in IR-resistant A172 cells and significantly in Ln229 cells and reduced clonogenic survival after irradiation only on these two cell lines. Although USP9x knockdown reduced Mcl-1 levels and increased apoptosis in A172 cells, USP9x regulated radiosensitivity independently of Mcl-1 in Ln229 cells.

Our results show a different requirement of USP9x in the control of glioblastoma cell survival and radiosensitivity.

## Results

### Mcl-1 and USP9x are upregulated during tumor progression

**Figure 1:**
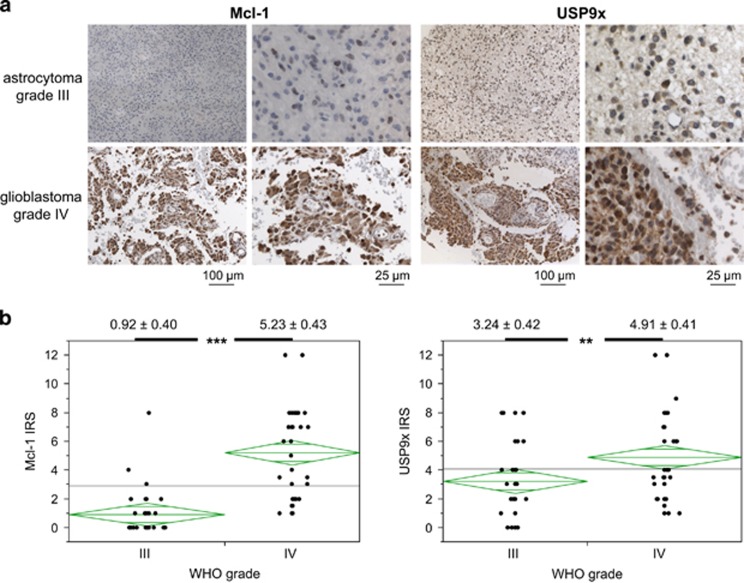
Mcl-1 and USP9x expression was increased in highly malignant glioblastoma. Mcl-1 and USP9x protein expressions were examined in human astrocytoma (WHO grade III) and glioblastoma (WHO grade IV) tissue samples by immunohistochemical staining. (**a**) Representative tissue samples stained with Mcl-1 or USP9x antibodies are shown in two magnifications. (**b**) Staining intensity and quantity were analyzed, and the combined immunoreactive score (IRS) was calculated for Mcl-1 (right panel) and USP9x (left panel). Median IRS±S.D. is shown above the respective group and staining. **P<0.01, ***P<0.001. Mcl-1 and USP9x expressions were significantly higher in glioblastoma than in astrocytoma

In the first set of experiments, we examined the expression of Mcl-1 and USP9x in astrocytoma (WHO grade III) and glioblastoma (WHO grade IV) (Figure 1). Immunohistochemical analysis shows that number of Mcl-1- and USP9x-positive cells and staining intensity were significantly upregulated in glioblastoma compared with astrocytoma (Figure 1b). Median immune reactivity score (IRS) of Mcl-1 increased from 0.92±0.40 (95% from 0.13 to 1.71, *n*=38) in grade III astrocytoma to 5.23±0.43 (95% from 4.38 to 6.08, *n*=33) in glioblastoma, whereas IRS of USP9x increased from 3.24±0.42 (95% from 2.39 to 4.08, *n*=42) in astrocytoma to 4.91±0.41 (95% from 4.10 to 5.73, *n*=45) in glioblastoma. A more detailed mosaic plot analysis of Mcl-1 and USP9x IRS shows that Mcl-1 was upregulated in more glioblastoma tissue samples and to a greater extent than USP9x (Supplementary Figure S1). Yet, Mcl-1 and USP9x IRS correlated moderately but significantly in grade IV glioblastoma (Spearman correlation, *ρ*=0.47, *P*=0.0063), indicating a coincidental upregulation of Mcl-1 and USP9x.

### IR-induced downregulation of Mcl-1 correlates with apoptosis induction

**Figure 2:**
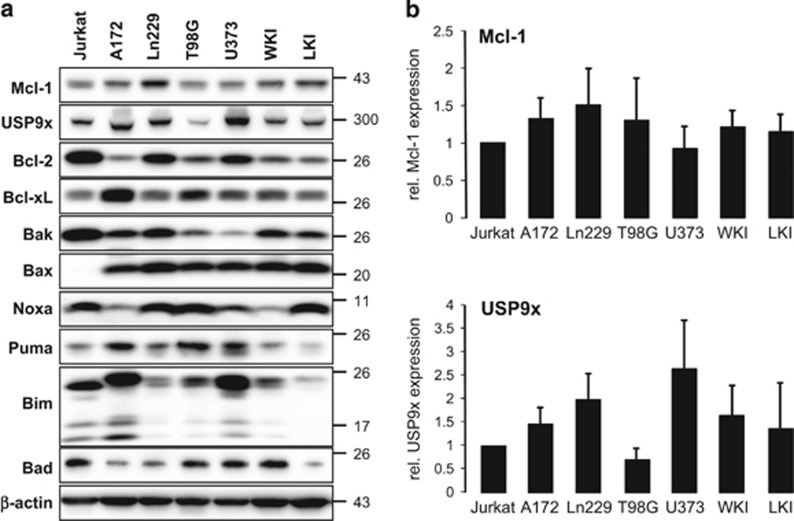
Protein levels of different Bcl-2 family members and the deubiquitinase USP9x in glioblastoma cell lines. (**a**) Cell lysates of established glioblastoma cell lines A172, T98G, U373, and Ln229 as well as primary glioblastoma cells WKI and LKI and Jurkat T-lymphoma cells were separated by gel electrophoresis. Protein levels of the antiapoptotic Mcl-1, Bcl-2, Bcl-xL, and the pro-apoptotic Bax, Bak, Noxa, Puma, Bim, and Bad were analyzed by western blot. In addition, protein levels of the Mcl-1-stabilizing deubiquitinase USP9x were determined in all cell lines. *β*-actin was used as loading control. Mcl-1 and USP9x protein levels were higher in glioblastoma cells than in Jurkat T-lymphoma cells. (**b**) Densitometric analysis of USP9x and Mcl-1 was performed. Protein levels of Mcl-1 (upper panel) and USP9x (lower panel) were normalized to *β*-actin and then to these of Jurkat T cells. Results show mean values±S.D. (*n*=3). No significant regulation was observed

Previous experiments have shown the effect of USP9x on Mcl-1 in irradiated Jurkat lymphoma cells.^9^ Thus, we compared the protein levels of USP9x, Mcl-1 and several other Bcl-2 family members in Jurkat cells, four established (A172, Ln229, T98G, U373), and two primary (WKI, LKI) glioblastoma cell lines (Figure 2). All four glioblastoma cell lines expressed USP9x as well as antiapoptotic Mcl-1, Bcl-2, Bcl-xL, and pro-apoptotic Bax, Bak, Noxa, Puma, and Bad (Figure 2a). The protein levels of USP9x and Mcl-1 were slightly but insignificantly higher in five of six glioblastoma cell lines than in Jurkat cells (Figure 2b). Although all glioblastoma cell lines expressed pro-apoptotic Bax at similar levels, the levels of the other pro- and antiapoptotic proteins greatly differed between the cell lines (Figure 2a).

**Figure 3:**
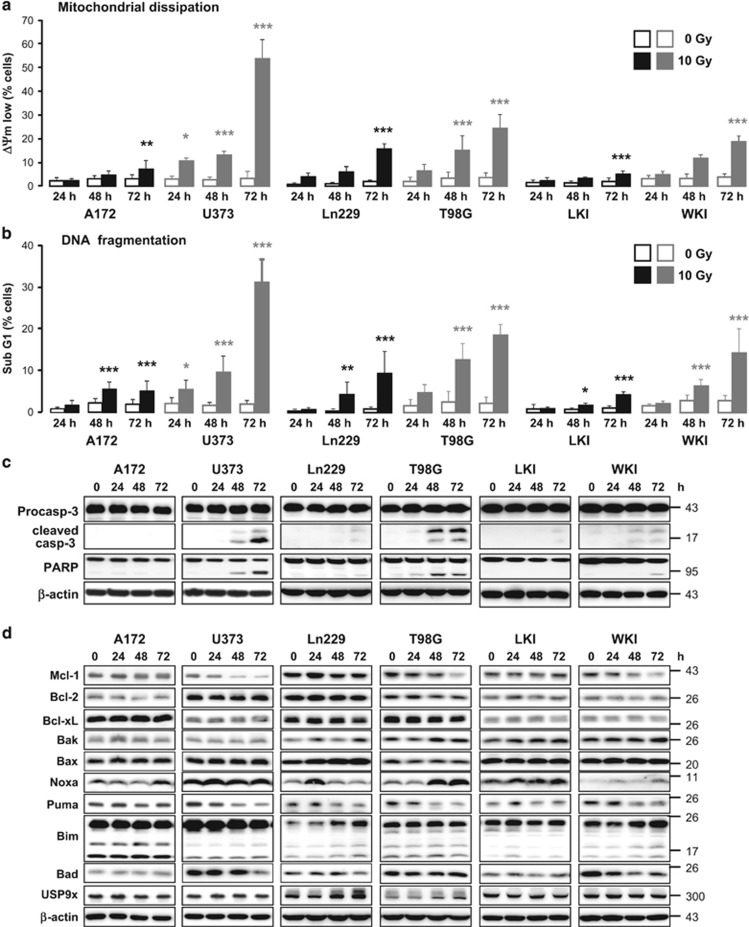
Ionizing radiation induced downregulation of Mcl-1 and caspase-dependent apoptosis in U373, T98G, and WKI cells. A172, U373, Ln229, T98G, LKI, and WKI glioblastoma cells were irradiated with 0 Gy or 10 Gy. 24 h, 48 h, and 72 h after irradiation, dissipation of mitochondrial membrane potential (ΔΨm low, **a**) and DNA fragmentation (sub G1, **b**) were analyzed by flow cytometry. 72 h after irradiation, dissipation of ΔΨm and DNA fragmentation were clearly increased in U373, T98G, and, to a lesser extent, in WKI cells. Flow cytometric data show mean values±S.D. (*n*≥6). Significance was calculated to the respective non-irradiated control cells. *P<0.05, **P<0.01, ***P<0.001. (**c**, **d**) 0–72 h after irradiating glioblastoma cells, lysates were made and analyzed by western blot. *β*-actin was used as loading control. (**c**) Caspase-3 and PARP cleavage were detected only in U373, T98G, and WKI cells 48 and 72 h after irradiation, indicating apoptosis induction in response to IR in these cells but not in A172, Ln229, and LKI cells. (**d**) Protein levels of antiapoptotic Mcl-1 were downregulated in U373, T98G, and WKI cells 48 and 72 h after irradiation. The Mcl-1 decay correlated with cpase-3 and PARP cleavage. No change of antiapoptotic Bcl-2 and Bcl-xL levels was observed upon irradiation. The regulation of the pro-apoptotic proteins in response to IR differed between the cell lines, but it did not correlate with apoptosis induction

Next, we irradiated glioblastoma cells with 10 Gy and determined dissipation of mitochondrial membrane potential (ΔΨm) and DNA degradation (sub G1 population) by flow cytometry (Figure 3a and b) to analyze radiation-induced cell death and apoptosis, respectively. Although ΔΨm dissipation and DNA fragmentation were hardly increased in A172, Ln229, and LKI cells in response to IR, irradiation effectively increased the cell population with dissipated ΔΨm and fragmented DNA in a time-dependent manner in U373 cells and, to a lesser extent, in T98G and WKI cells. Similar results were obtained by measuring cell death using an exclusion dye assay (Supplementary Figure S3).

Western blot analysis clearly showed caspase-3 and PARP cleavage in U373 and T98G cells and weaker caspase-3 and PARP cleavage in WKI cells, indicating that IR induced caspase-dependent apoptosis in U373, T98G, and WKI cells but not in A172, Ln229, and LKI cells (Figure 3c).

As members of the Bcl-2 protein family regulate the mitochondrial homeostasis and the intrinsic apoptosis pathway in response to IR,^1, 14^ up- and downregulation of those proteins might be responsible for the IR-induced apoptosis in the three glioblastoma cell lines. Therefore, we analyzed the protein levels of different Bcl-2 family members in response to IR (Figure 3d). Although levels of antiapoptotic Bcl-2 and Bcl-xL did not change after irradiation in all six cell lines, IR-induced downregulation of Mcl-1 correlated with caspase-3 activation in U373, T98G, and WKI cells. Levels of the Mcl-1-regulating deubiquitylating enzyme USP9x were only slightly increased by IR in Ln229 cells and not affected in the other five cell lines. The pro-apoptotic Bcl-2 family members were regulated differently in response to IR in the six glioblastoma cell lines, but their regulation did not correlate with apoptosis induction.

### IR reduced Mcl-1 stability in U373 cells

**Figure 4:**
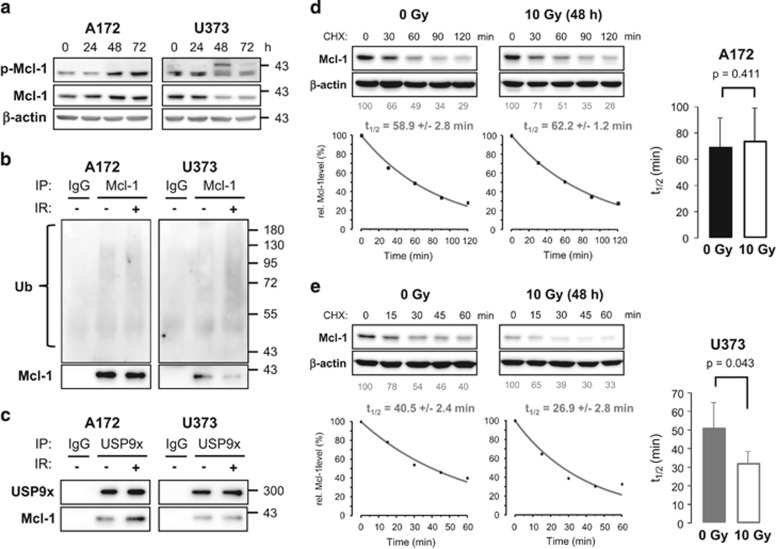
Mcl-1 stability decreased in U373 but not in A172 cells following irradiation. (**a**) 0–72 h after irradiating A712 and U373 cells with 10 Gy, lysates were made. Phospho (S163)-Mcl-1 and total Mcl-1 were analyzed by western blot. *β*-actin was used as loading control. A shift of phosphorylated Mcl-1 was observed in U373 cells 48–72 h after irradiation, indicating post-translational modification of Mcl-1. (**b**, **c**) 48 h after irradiating A172 and U373 cells with 0 or 10 Gy (IR:- or +), lysates were made. (**b**) Mcl-1 was precipitated (IP: Mcl-1), and the ubiquitylation status of Mcl-1 was analyzed by western blot. (**c**) USP9x was precipitated (IP: USP9x), and USP9x as well as co-precipitated Mcl-1 were analyzed by western blot. As a control, precipitation experiments were performed with unspecific isotype-matched immunoglobulins (IP: IgG) and lysates from non-irradiated cells. Ubiquitylated Mcl-1 was increased 48 h after IR in U373 but not in A172 cells. At the same time, more Mcl-1 precipitated with USP9x in A172 cells after irradiation, whereas the association was not changed in U373 cells. Precipitation experiments were performed twice. After 48 h of irradiation with 0 Gy or 10 Gy, A172 (**d**) and U373 (**e**) cells were treated with 5 *μ*M cycloheximide (CHX) for the indicated time (upper panels). Cells were lysed at respective time points, and Mcl-1 levels were analyzed by western blot. *β*-actin was used as loading control. After densitometric quantification, values were normalized to the respective loading control (*β*-actin) and then to the respective untreated control values. The results of the densitometric analysis are shown below the blots (lower panels). Subsequently, Mcl-1 values were fitted and the half-life of Mcl-1 was calculated (right panels). Bar diagrams show the average half-life time of Mcl-1 in non-irradiated and irradiated cells±S.D. (*n*=3). Mcl-1 stability remained unchanged in A172 cells but was significantly decreased in U373 cells 48 h after irradiation

We selected IR-resistant A172 cells and IR-sensitive U373 cells to further analyze Mcl-1 ubiquitylation and stability. As phosphorylation of Mcl-1 at serine 163 is associated with an accelerated degradation rate, the phosphorylation status was analyzed by western blot (Figure 4a). Increased phospho-Mcl-1 levels were detected in A172 cells 48–72 h after IR. In contrast, the 40 kDa form of phosphorylated Mcl-1 decreased in U373 cells 48–72 h following irradiation, but a shift of phosphorylated Mcl-1 was clearly visible, indicating a post-translational modification. Such a modification can be ubiquitylation, which has been associated with an increased degradation rate before.^10, 11^ Thus, we precipitated Mcl-1 to analyze its ubiquitylation by western blot (Figure 4b). Mcl-1 ubiquitylation was not affected in A172 cells, but was clearly increased in U373 cells 48 h after irradiation. Further experiments showed that more Mcl-1 co-precipitated with USP9x in A172 cells after irradiation, whereas an increased association of Mcl-1 with USP9x was not observed in irradiated U373 cells (Figure 4c). Levels of Mcl-1, USP9x, and ubiquitylated proteins in whole-cell lysates that were used for both precipitation experiments are shown in supplementary figure (Supplementary Figure S5A).

We also analyzed the protein stability of Mcl-1 in A172 and U373 cells 48 h after irradiation and in non-irradiated cells. Mcl-1 was degraded at similar rates in non-irradiated and irradiated A172 cells (Figure 4d). In contrast, the half-life time significantly decreased in U373 cells after IR (Figure 4e). Moreover, comparing Mcl-1 stability in both non-irradiated cell lines, Mcl-1 was degraded slightly but insignificantly faster in U373 than in A172 cells (Supplementary Figure S5B).

Summarized, our results suggest that Mcl-1 stability is regulated by different mechanisms in irradiated A172 and U373 glioblastoma cells.

### Mcl-1 and USP9x affect cell viability in glioblastoma cells

**Figure 5:**
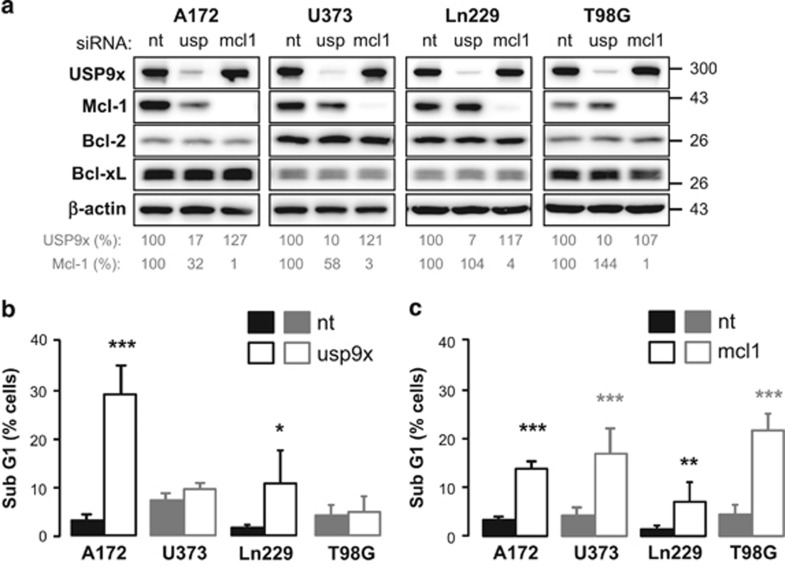
Downregulation of Mcl-1 induced apoptosis in all glioblastoma cells, whereas downregulation of USP9x induced apoptosis only in IR-resistant A172 and Ln229 cells. (**a**, **b**) A172, U373, Ln229, and T98G cells were transfected with 100 nM siRNA targeting USP9x (usp) or 50 nM siRNA-targeting Mcl-1 (mcl1). Control transfection was performed with respective amount of non-targeting (nt) siRNA. (**a**) After 72 h of transfection, protein levels of USP9x, Mcl-1, Bcl-2, and Bcl-xL were analyzed by western blot. *β*-actin was used as loading control. After densitometric analysis, USP9x and Mcl-1 protein levels were normalized to the respective loading control followed by normalization to respective protein levels in cells transfected with nt siRNA. USP9x knockdown decreased Mcl-1 protein levels clearly in A172 and slightly in U373 cells. After 72 h of transfection with USP9x siRNA (**b**), Mcl-1 siRNA (**c**), or the respective amount of nt siRNA, DNA fragmentation was analyzed by flow cytometry. USP9x knockdown resulted in significant apoptosis induction in A172 and Ln229 cells only (**b**), whereas Mcl-1 knockdown significantly induced apoptosis in all glioblastoma cell lines. (**c**) Flow cytometric data show mean values±S.D. (A172, U373: *n*=3; Ln229: *n*=5; T98G: *n*=10). Significance was calculated to the respective cells transfected with non-targeting siRNA. *P<0.05, **P<0.01, ***P<0.001

In previous publications, a stabilizing effect of deubiquitinase USP9x on Mcl-1 was described.^12, 15, 16^ Therefore, USP9x and Mcl-1 were downregulated by siRNA in glioblasoma cells (Figure 5). USP9x knockdown resulted in decreased Mcl-1 levels in A172 and less strikingly in U373 cells, but did not change Mcl-1 levels in Ln299 and T98G cells (Figure 5a). Neither Bcl-2 nor Bcl-xL levels were affected by transfection.

The lowered Mcl-1 levels might result in reduced cell survival due to increased apoptosis. To examine the effect of USP9x and Mcl-1 knockdown on apoptosis induction, DNA fragmentation was analyzed by flow cytometry 72 h following transfection. Downregulation of USP9x markedly increased the amount of A172 cells with fragmented DNA and to lesser extent of Ln229 cells, whereas it hardly induced DNA fragmentation in U373 and T98G cells (Figure 5b). In contrast, Mcl-1 knockdown resulted in significantly increased DNA fragmentation in all four cell lines (Figure 5c).

### Effect of USP9x and Mcl-1 knockdown on irradiated glioblastoma cells

We previously described a radiosensitizing effect of USP9x in Jurkat cells.^9^ Therefore, we examined the influence of Mcl-1 and USP9x knockdown on apoptosis induction in glioblastoma cells following irradiation with 10 Gy by flow cytometry analyzing DNA fragmentation and ΔΨm dissipation.

**Figure 6:**
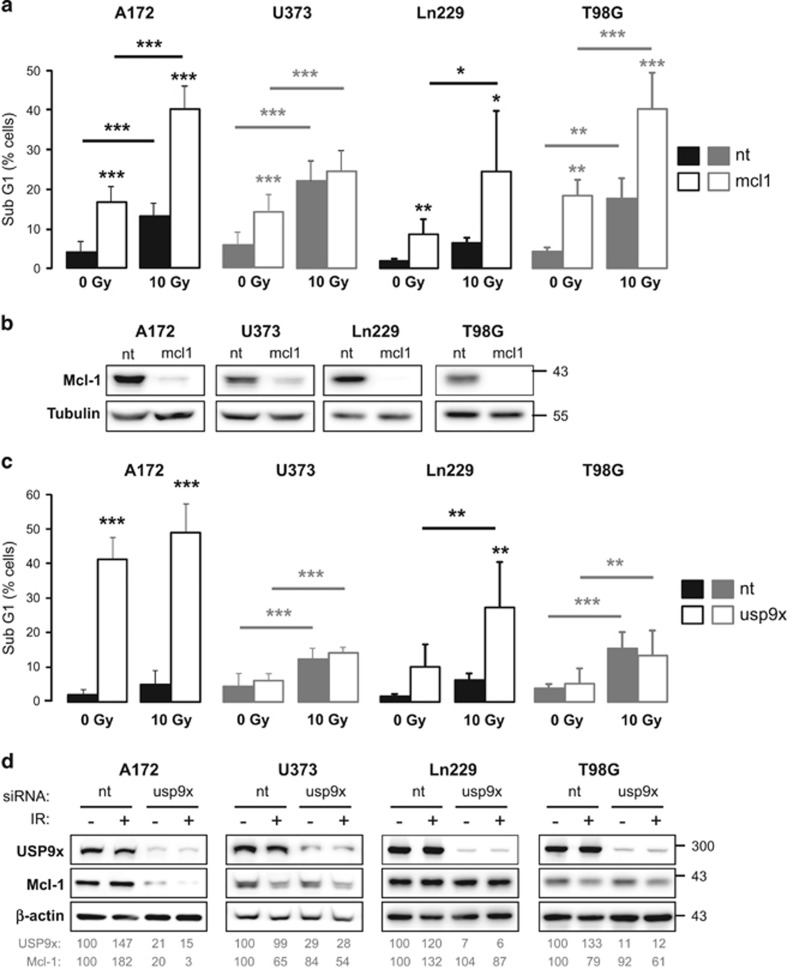
Effects of Mcl-1 and USP9x knockdown on IR-induced apoptosis in glioblastoma cells. (**a**–**d**) A172, U373, Ln229, and T98G cells were transfected with 50 nM siRNA targeting Mcl-1 (mcl1), 100 nM siRNA targeting USP9x (usp9x), or with the respective amount of non-targeting (nt) siRNA. After 48 h, cells were irradiated with 0 Gy or 10 Gy. (**a**) 48 h after irradiation, DNA fragmentation was analyzed by flow cytometry after Mcl-1 knockdown. Data shows mean values±S.D. (A172, U373: *n*=6; A172, Ln229, T98G: *n*=5). (**b**) Mcl-1 knockdown was verified by western blot 48 h after transfection. Mcl-1 knockdown significantly increased IR-induced DNA fragmentation in A172, Ln229. and T98G cells. (**c**) After 48 h of irradiation, DNA fragmentation was analyzed by flow cytometry after USP9x knockdown. Data show mean values±S.D. (A172: *n*=6; U373: *n*=9; Ln229, T98G: *n*=5). (**b**) USP9x and Mcl-1 protein levels were analyzed by western blot. *β*-actin was used as loading control. After densitometric analysis, USP9x and Mcl-1 levels were normalized to respective *β*-actin levels and then to non-irradiated cell transfected with nt siRNA. USP9x knockdown slightly enhanced IR-induced downregulation of Mcl-1 levels and apoptosis induction in A172 cells, whereas, in Ln229 cells, IR-induced apoptosis was significantly increased without affecting Mcl-1 levels. Significance in **a** and **c** was calculated to the respective cells transfected with nt siRNA and, where indicated by a line, to non-irradiated control cells. *P<0.05, **P<0.01, ***P<0.001

Downregulation of Mcl-1 significantly increased IR-induced DNA fragmentation in A172, Ln229, and T98G cells, but had no effect in U373 cells (Figure 6a). Successful Mcl-1 knockdown in glioblastoma cells was verified by western blot (Figure 6b). IR-induced ΔΨm dissipation was increased in all four cell lines transfected with Mcl-1 siRNA (Supplementary Figure S6A). The data suggest that lowered Mcl-1 levels could sensitize the glioblastoma cells to IR-induced apoptosis or, in case of U373 cells, accelerate IR-induced apoptosis.

In A172 cells, downregulation of USP9x by siRNA already resulted in intense DNA fragmentation that was insignificantly increased after irradiation (Figure 6c). A significant increase of IR-induced DNA fragmentation was observed in Ln229 cells transfected with USP9x siRNA. In contrast, no effect of USP9x knockdown on IR-induced DNA fragmentation was observed in U373 and T98G cells. Similar effects were observed by analyzing ΔΨm dissipation (Supplementary Figure S6B). The data show that USP9x knockdown could sensitize Ln229 cells to IR-induced apoptosis or accelerate IR-induced apoptosis as in U373 cells, but had no effect in other glioblastoma cell lines.

Following transfection with USP9x siRNA and irradiation, we observed a strong downregulation of Mcl-1 in non-irradiated A172 cells, which was even stronger after irradiation (Figure 6d). In contrast, IR resulted in decreased Mcl-1 levels in U373 and T98G cells, but the Mcl-1 protein level was not affected by USP9x knockdown. In Ln229 cells, neither USP9x siRNA nor irradiation had any effect on Mcl-1.

Taken together, downregulation of Mcl-1 could sensitize glioblastoma cells to or accelerate IR-induced apoptosis. In contrast, downregulation of USP9x had a strong effect on cell survival in A172 cells but showed a sensitizing effect to IR-induced apoptosis only in Ln229 cells without affecting Mcl-1 levels.

### Irradiation and Mcl-1 knockdown sensitized glioblastoma cells to Bcl-2/Bcl-xL-induced apoptosis

**Figure 7:**
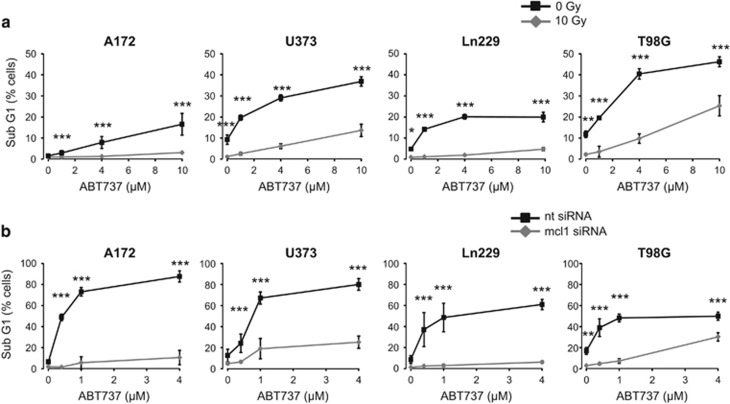
Inhibition of Bcl-2 and Bcl-xL improved apoptosis induction in response to irradiation and downregulation of Mcl-1. (**a**) A172, U373, Ln229, and T98G cells were irradiated (0 Gy, 10 Gy) and treated with the Bcl-2/Bcl-xL inhibitor ABT737 (0 *μ*M/solvent control, 1, 4, or 10 *μ*M) immediately after irradiation. DNA fragmentation was analyzed 48 h after irradiation and treatment. IR increased ABT737-induced apoptosis in all glioblastoma cells, but to a greater extent in U373 and T98G than in A172 and Ln229 cells. Flow cytometric data show mean values±S.D. (A172, U373: *n*=6; Ln229, T98G: *n*=3). (**b**) A172, U373, Ln229, and T98G cells were transfected with 50 nM Mcl-1 (mcl1) siRNA or the respective non-targeting (nt) siRNA. After 24 h of transfection, cells were treated with ABT737 (0 *μ*M/solvent control, 0.4, 1, or 4 *μ*M). After 48 h, DNA fragmentation was analyzed by flow cytometry. Mcl-1 knockdown facilitates ABT737-induced apoptosis in A172 and Ln229 cells to a similar extent as in U373 and T98G cells. Flow cytometric data show mean values±S.D. (*n*=3). Significance was calculated to the respective non-irradiated cells. *P<0.05, **P<0.01, ***P<0.001

Maintaining high Mcl-1 levels seemed to be important for all glioblastoma cells. However, all antiapoptotic Bcl-2 family members need to be neutralized to successfully induce apoptosis. To analyze the role of other antiapoptotic Bcl-2 family members in the control of cell survival, we treated A172, U373, Ln299, and T98G cells with different concentrations of the Bcl-2/Bcl-xL inhibitor ABT737 and with radiotherapy. Apoptosis was assessed by flow cytometry analyzing DNA fragmentation (Figure 7a) and ΔΨm dissipation (Supplementary Figure S7A). Our results show that U373 and T98G cells reacted more susceptible to ABT737 than A172 and Ln229 cells. However, combined with radiotherapy, ABT737 increased apoptosis in all four glioblastoma cell lines. Moreover, our data show that irradiated cells that downregulate Mcl-1 levels respond better to Bcl-2/Bcl-xL inhibition than irradiated cells that fail to downregulate Mcl-1. To confirm this observation described above, we knocked down Mcl-1 by siRNA before treating the cells with ABT737 (Figure 7b, Supplementary Figure S7B). As expected, Mcl-1 knockdown sensitized all four glioblastoma cell lines to ABT737-induced apoptosis. Apoptosis induction by Bcl-2/Bcl-xL inhibition was as effective in A172 and Ln229 cells as in U373 and T98G cells. These results confirm the importance of Mcl-1 in regulation of survival.

### USP9x knockdown increased sensitivity to IR in A172 and Ln229 cells

**Figure 8:**
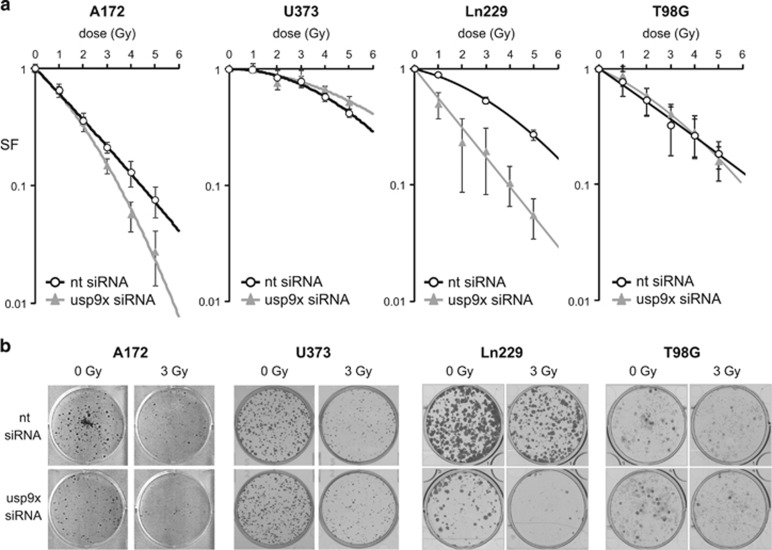
Downregulation of USP9x by siRNA increased clonogenic death of irradiated A172 and Ln229 cells. In total, 3000 cells were seeded per well and transfected with 100 nM siRNA-targeting USP9x (usp9x) or non-targeting (nt) siRNA 24 h later. After 48 h of transfection, A172 and U373 cells were irradiated with 0–5 Gy. Colonies were allowed to form for 8–10 days. After counting the colonies, the surviving fraction (SF) was calculated. Surviving curves in response to IR are shown in (**a**), whereas representative wells are shown in (**b**). USP9x knockdown decreased the respective surviving fractions and, thus, increased clonogenic cell death in A172 and Ln229 cells, but showed no significant effect in U373 cells. Data show mean SF values±S.D. of one experiment that was performed in triplicates. One representative experiment of two is shown

After measuring the influence of USP9x on irradiated glioblastoma cells in a short-term assay, we analyzed the influence of USP9x on long-term survival by a colony-formation assay measuring the clonogenic survival upon transfection with USP9x siRNA or the non-targeting control siRNA and irradiation (Figure 8). After irradiation, the surviving fraction (SF) was decreased in all four glioblastoma cell lines in a dose-dependent manner. USP9x knockdown further reduced SF in A172 and Ln229 cells. In contrast, USP9x did not affect radiosensitivity in U373 and T98G cells. Our data show that USP9x regulates radiosensitivity only in some glioblastoma cells.

## Discussion

Patients with glioblastoma usually undergo neurosurgical tumor removal before adjuvant radiotherapy, usually combined with temozolomide.^13^ However, many patients do not respond or respond only partially to IR. Novel strategies are needed to improve the response to radiochemotherapy. One therapeutic option might be the targeting of the antiapoptotic Bcl-2 family members that control intrinsic apoptosis.

Here, we used four established glioblastoma cell lines and two primary glioblastoma cell lines that express the antiapoptotic proteins Bcl-2, Bcl-xL, and Mcl-1, but showed different sensitivity to IR-induced apoptosis. In contrast to the A172, Ln229 and LKI cells, the cell lines U373, T98G, and WKI downregulated Mcl-1 and induced caspase-dependent apoptosis in response to IR. The decline of Mcl-1 levels upon irradiation have been correlated before with apoptosis induction in Jurkat T-lymphoma cells.^9^ In addition, Mcl-1 increased resistance of glioblastoma cells to apoptosis induced by temozolomide or the death ligand TRAIL.^17, 18^ Thus, the failure to downregulate Mcl-1 levels in A172, Ln229, and LKI cells could be responsible for survival after irradiation.

### Regulation of Mcl-1 stability in glioblastoma cells

Mcl-1 levels are regulated at the transcriptional and translational level.^8, 19^ Moreover, Mcl-1 stability is controlled by post-translational modifications. Usually, Mcl-1 is quickly ubiquitylated and subsequently degraded by proteasomes. Until now, three ubiquitin ligases have been described that catalyze the ubiquitylation of Mcl-1,^11, 20, 21, 22^ whereas the deubiquitinase USP9x is able to antagonize this reaction by removing the polyubiquitin chains, thereby stabilizing Mcl-1.^9, 21^ In addition, phosphorylation of Mcl-1 by GSK-3*β* can accelerate Mcl-1 ubiquitylation and degradation.^10^ Our results show that phosphorylated Mcl-1 was more ubiquitylated, whereas Mcl-1 half-life time was reduced in U373 cells after irradiation. Neither Mcl-1 ubiquitylation nor Mcl-1 stability were affected in A172 cells in response to irradiation. The data suggest that, in U373 cells, ubiquitylation targeted Mcl-1 for proteasomal degradation in response to IR, whereas phosphorylated Mcl-1 was stabilized in irradiated A172 cells. As the interaction between Mcl-1 and the deubiquitinase USP9x was not changed in U373 cells upon irradiation, we think that the increased Mcl-1 polyubiquitylation was due to enhanced ubiquitylation activity rather than to reduced USP9x activity. Alternatively, the activity of another, not yet identified, deubiquitinase regulating Mcl-1 stability in U373 cells could be compromised in U373 cells upon irradiation. Ku70, a component of the non-homologs end joining DNA repair pathway, was recently shown to interact with and deubiquitylate Mcl-1, thereby linking DNA repair to apoptosis.^23^ Whether the activity of Mcl-1-specific ubiquitin ligases is increased or the Ku70 deubiquitylating activity is reduced after IR in apoptosis-inducing glioblastoma cells, this needs further investigation. On the other hand, the increased interaction between Mcl-1 and USP9x in irradiated A172 cells implicates that USP9x contributes to Mcl-1 stabilization, thereby improving survival following irradiation in this cell line.

### USP9x is a potential radiosensitizing factor

Low USP9x levels have been correlated with low Mcl-1 levels and increased susceptibility to IR.^12^ Thus, knockdown of USP9x might result in cell death when cell survival depends on high Mcl-1 levels. In A172, downregulation of USP9x by siRNA resulted in lowered Mcl-1 levels and massive apoptosis induction. Irradiation only slightly increased cell death, whereas it further decreased Mcl-1 levels. Nevertheless, USP9x knockdown had a remarkable radiosensitizing effect in colony-formation assay. A radiosensitizing effect was also observed in Ln229 cells after transfection with USP9x siRNA. In this cell line, downregulation of USP9x resulted in increased apoptosis and reduced clonogenic survival. However, Mcl-1 protein levels were neither affected by USP9x nor by IR, suggesting that USP9x increased resistance to IR-induced apoptosis and clonogenic cell death independently of Mcl-1. In contrast, USP9x had no radiosensitizing effect in U373 and T98G cells, although USP9x knockdown accelerated IR-induced apoptosis in U373 cells.

USP9x has also been shown to positively regulate brain tumor growth.^24^ We detected a significantly higher USP9x expression in glioblastoma than in astrocytoma. Thus, our data indicate that USP9x has a role during tumor progression from astrocytoma to glioblastoma. USP9x expression correlated moderately but significantly with Mcl-1 expression in glioblastoma, supporting our hypothesis that USP9x stabilizes Mcl-1 in glioblastoma cells. However, not all glioblastoma tissue samples expressing high Mcl-1 levels also express high USP9x levels. This again implicates that other factors are also important regulators of Mcl-1 levels in glioblastoma tumors.

Further publications point to a multifaceted role of USP9x in the brain.^25, 26^ The diverse effects of USP9x could be signaled through different proteins targeted by USP9x. In addition to Mcl-1, USP9x also stabilizes *β*-Catenin and ubiquitin ligase Itch.^27, 28^ High *β*-catenin levels were correlated with increased radioresistance in pancreatic cancer cells,^29^ whereas Itch regulates the internalization of epidermal growth factor receptor, a growth receptor that mediates radioresistance in glioblastoma tumors.^30, 31^ Moreover, stabilization of Foxo3A by USP9x resulted in decreased cyclin D1 levels and cell cycle arrest.^32^ Although the three USP9x interaction partners have not been examined in our glioblastoma cells, it is possible that USP9x modulates the response to IR in Ln229 and A172 cells through these and other effector proteins.

### Dependency on Bcl-2/Bcl-xL

The antiapoptotic family members Bcl-2, Bcl-xL, and Mcl-1 are often overexpressed in glioblastoma.^33^ Successful targeting of the protective proteins alone or in combination with other therapies has been repeatedly described.^5, 14, 34, 35, 36^ Generally, the more antiapoptotic proteins can be neutralized, the better is apoptosis induction. Among the best described inhibitors targeting antiapoptotic Bcl-2 family members is the Bad-mimicking compound ABT737 and its orally available analog ABT263, both of which were shown to inhibit Bcl-2 and Bcl-xL.^7, 37^ We have shown that A172 and Ln229 cells that hardly induced apoptosis after irradiation were also very resistant to ABT737-induced apoptosis, whereas U373 and T98G cells that induced apoptosis after irradiation reacted more sensitively to Bcl-2/Bcl-xL inhibition. The differences between the cell lines could be explained by different capacity to sequester pro-apoptotic Bcl-2 family members. Mcl-1 contributes to the neutralizing capacity, as downregulation of Mcl-1 sensitized all four glioblastoma cells to ABT737-induced apoptosis.

Interestingly, all cell lines could also be sensitized to ABT737-induced apoptosis by irradiation, suggesting additional sensitizing events by IR. Following irradiation, an upregulation of pro-apoptotic Bax was observed in Ln229 cells, whereas BH3-only protein Bim levels were increased in Ln229 and WKI cells in response to IR. Moreover, irradiation increased Noxa levels especially in T98G and WKI cells. Bim can antagonize all three antiapoptotic proteins and directly activate Bax and Bak.^38^ Noxa was described to specifically antagonize Mcl-1 but not Bcl-2 and Bcl-xL.^38^ In addition, a recent publication showed that Noxa is also able to directly activate Bax and Bak.^39^ All three proteins can shift the balance between anti- and pro-apoptotic Bcl-2 family members toward cell death. Interestingly, Bax and Noxa can be transcriptionally upregulated by p53, a tumor suppressor that is upregulated in response to IR.^40, 41, 42^ Alterations of p53 are commonly observed in glioblastoma.^43^ We found that p53 levels were already elevated in non-irradiated T98G, U373, and WKI cells, suggesting that p53 was mutated in these cell lines (Supplementary Figure S2). Moreover, none of the other glioblastoma cell lines induced p53 after irradiation, implicating an impaired p53 response following irradiation, thus, excluding any p53-dependent regulation of pro-apoptotic Bcl-2 family members.

Taken together, downregulation of Mcl-1 in response to IR is an important step in IR-induced apoptosis in glioblastoma cells. Furthermore, USP9x can act as a radioprotective protein either by maintaining high Mcl-1 levels or by regulating alternative mechanisms.

## Material and Methods

### Reagents and antibodies

Cycloheximide was purchased from Sigma (Deisenhofen, Germany). ABT737 was obtained from Active Biochemicals (Bonn, Germany).

Following antibodies were used for western blotting: rabbit-anti Bak from Upstate (distributed by Millipore, Schwalbach, Germany), rabbit- anti caspase-3, PARP, Mcl-1, Bcl-xL, Bax, Puma, Bim, Tubulin, and mouse anti-Ubiquitin (clone P4D1) from Cell Signaling (distributed by NEB, Frankfurt, Germany), mouse anti–Mcl-1 from Pharmingen and mouse anti-Bad from Transduction Laboratories (distributed by Becton Dickinson, Heidelberg, Germany), mouse-anti Bcl-2 from Santa Cruz Biotechnology (Heidelberg, Germany), mouse anti-Noxa from Calbiochem (distributed by Merck, Darmstadt, Germany), rabbit-anti-USP9x from Novus Biologicals (distributed by Acris, Herford, Germany), and mouse-anti *β*-actin was obtained from Sigma.

### Cells and cell culture

A172, Ln229, U373, and T98G glioblastoma cell lines as well as Jurkat T-lymphoma cells were from ATCC (Bethesda, MA, USA). According to ATCC, U373 cells used in the present work show genetic similarities to U251 glioblastoma cells. LKI cells were established from primary glioblastoma and provided by the Department of Radiation Oncology, University of Tuebingen, Germany, with patient's consent and approved by the local ethic committee (579/2015BO2), whereas WKI cells were provided by Dr. Mike Fay from Genesis CancerCare (New South Wales, Australia). Cells were grown in RPMI 1640 medium supplemented with 10% fetal bovine serum (Gibco Life Technologies, Eggenstein, Germany). Cells were maintained in a humidified incubator at 37 °C and 5% CO_2_.

Cells were irradiated at room temperature with 6 MV photons using a linear accelerator (LINAC SL25 Philips, DA Best, the Netherlands) at a dose rate of 4 Gy/min.

### Transfection with siRNA

In total, 3–4 × 10^5^ cells were seeded in 2 ml complete medium (RPMI 1640+10% fetal bovine serum) in a six-well plate. After 24 h, cells were transfected with 50–100 nM of the respective siRNA using Trans-IT siQuest transfection reagent (Mirus, Madison, WI, USA) according to the manufacturers protocol. Mcl-1 and USP9x ON-TARGET SMARTpools, and siCONTROL NON-TARGETING pool were purchased from Dharmacon (Chicago, IL, USA).

### Flow cytometric analysis

The mitochondrial membrane potential (ΔΨm) was analyzed using the ΔΨm-specific dye tetramethylrhodamine ethyl ester (TMRE, Molecular Probes, distributed by Thermo Fisher Scientific, Grand Island, NY, USA). At the indicated time points, cells were stained for 30 min in PBS containing 25 nM TMRE. To examine DNA fragmentation, cells were incubated with PBS containing 0.1% sodium citrate, 0.1% Triton X-100, and 10 *μ*g/ml propidium iodide. Cells were detected in fluorescence channel 2 employing a FACS Calibur flow cytometer (Becton Dickinson) and analyzed with the FCS Express 3 software (De Novo Software, Los Angeles, CA, USA). Data show mean values±S.D. of at least six independent experiments.

### Colony-formation assay

Clonogenic survival was analyzed as described before.^44^ In brief, 3000 cells were seeded in six-well plates and transfected with 100 nM USP9x or non-targeting siRNA the next day. After 24 h of transfection, cells were irradiated with 0–5 Gy. The cells were incubated as described above for 6–10 days (depending on the cell line) to allow growth of single colonies. After that, cells were fixed with 3.7% formaldehyde and 70% ethanol and subsequently stained with 0.05% Coomassie Brilliant Blue. Colonies (>50 cells/colony) were counted. To determine the SF, the ratio of colonies counted/seeded cells was calculated and normalized to that of untreated control cells. The fitting of the curves was performed using Excel software. Error bars indicate the mean values±S.D. Two independent experiments were performed.

### Western blot analysis

Cells were lysed in 200 *μ*l lysis buffer containing 50 mM HEPES pH 7.5, 150 mM NaCl, 1% Triton X-100, 1 mM EDTA, 10 mM sodium pyrophosphate, 10 mM NaF, 2 mM Na_3_VO_4_, 100 mM PMSF, 5 *μ*g/ml Aprotinin, 5 *μ*g/ml Leupeptin, and 3 *μ*g/ml Pepstatin A. Protein was separated by SDS-PAGE under reducing conditions and transferred onto PVDF membranes (Roth, Karlsruhe, Germany). Blots were blocked in TBS buffer containing 0.05% Tween 20 and 5% non-fat dry milk for 1 h at room temperature. The membrane was incubated overnight at 4 °C with the respective primary antibodies. The secondary antibody was incubated for 1 h at room temperature. Detection of antibody binding was performed by enhanced chemoluminescence (ECL Western blotting analysis system from GE Healthcare, Freiburg, Germany). Equal loading was verified by antibodies against *β*-actin or Tubulin. Where indicated protein levels were quantified by densitometry using ImageJ software (ImageJ 1.40 g NIH, USA). At least two independent western blot experiments were performed.

### Mcl-1 degradation assay

Cells were treated with 2 *μ*M cycloheximide for 0–3 h. At indicated time points, cells were lysed, and lysates were separated as described above. Mcl-1 protein levels were detected by western blot. Several blots were made from the same lysates. Protein levels were quantified by densitometry using ImageJ software (ImageJ 1.40 g NIH, USA) and normalized to the *β*-actin levels. After that, Mcl-1 levels were normalized to the initial level (0 min cycloheximide). Monoexponential decay was fitted using Origin 6.0 software and included in further analysis when correlation coefficient *R*^2^ was higher than 0.8. At least five western blots were analyzed of each experiment and the median was calculated. Three independent experiments were performed.

### Immunoprecipitation

Cells were lysed as described above using 1% CHAPS as detergent. The protein concentration was adjusted to 2 mg/ml. Two micrograms mouse anti-USP9x antibody (H00008239-M01, Abnova, Acris) or five rabbit anti-Mcl-1 (S19, Santa Cruz Biotechnology) and 50 *μ*l slurry Dynabeads suspension (Dynal/Invitrogen, Karlsruhe, Germany) were added to 750 *μ*l lysate. After the precipitation for 3 h at 4 °C, the beads were washed thrice with 300 *μ*l lysis buffer containing 0.2% of the respective detergent. Proteins were eluted by boiling the beads for 10 min in 60 *μ*l SDS sample buffer containing 2.5% *β*-mercaptoethanol. 30 *μ*l of the eluate were separated by SDS gel electrophoresis before transfer to PVDF membrane and detection by chemoluminescence as described above. Precipitations were performed twice in two independent experiments.

### Immunohistochemistry

Human tissue samples were obtained from the department of Neurosurgery, University of Tuebingen, with patients' consent approved by the local ethic committee (163/2012B02). Formalin-fixed, paraffin-embedded human tumor specimens (astrocytoma WHO grade III, *n*=42; glioblastoma, WHO grade IV, *n*=45) were placed on tissue microarrays (TMA, two cores, 1000 *μ*m each) under supervision of a neuropathologist. TMA blocks were cut to 4mm slides and deparaffinized. Immunohistochemical stainings were performed using mouse anti-USP9x from Abnova (H00008239-M01, Acris, 1 : 800 dilution) and rabbit anti-Mcl-1 (S19, Santa Cruz Biotechnology, 1 : 1600 dilution) antibodies on the automated Benchmark immunohistochemistry system (Ventana Medical Systems, distributed by Roche Diagnostics, Mannheim, Germany). Heat-induced antigen retrieval was performed with CC1 cell conditioning solution (Tris-based EDTA buffer, Ventana) for 30 min for USP9x and no pretreatment for Mcl-1. Visualization of the specific antibody binding was achieved using the UltraView Universal DAB kit (Ventana). Human tonsils served as positive controls. Appropriate negative controls (omission of the first antibody) were processed in parallel with each batch of staining.

Staining evaluation: cytoplasmic and nuclear staining were evaluated together. Tumors were considered positive when >1% of the tumor cells exhibited a detectable immunoreactivity. The staining intensity was semiquantitatively recorded as absent, weak, moderate, and strong positive. The percentage of stained tumor cells was counted as 0 (negative), 1 (1–25%), 2 (26–50%), 3 (51–75%), and 4 (76–100%). Staining intensity score and the score indicating the amount of positive cells were multiplied to obtain the IRS ranging from 0 to 12. The median±S.D. and the 95 percentile were calculated for each grade and each antibody staining. To analyze the correlation between Mcl-1 IRS and USP9x IRS, Spearman correlation coefficient *ρ* was calculated after generating contingency tables for each WHO grade (JMP 11, Cary, NJ, USA).

### Data analysis

Statistical significance was calculated by student *t*-test or one-way ANOVA test followed by a Bonferroni post-test where appropriate using GraphPad Software (San Diego, CA, USA).

## Abbreviations

Bcl-2/-xL: B-cell leukemia-2/xL
BH3: Bcl-2 homology domain 3
GSK-3*β*: glycogen synthase kinase GSK-3*β*
IR: ionizing radiation
Mcl-1: myeloid cell leukemia sequence 1
Mule: Mcl-1 ubiquitin ligase E3
*β*-TrCP: *β*-transducin repeat-containing protein
USP9x: ubiquitin-specific protease 9x

## References

[bib1] Belka C, Rudner J, Wesselborg S, Stepczynska A, Marini P, Lepple-Wienhues A et al. Differential role of caspase-8 and BID activation during radiation- and CD95-induced apoptosis. Oncogene 2000; 19: 1181–1190. 10713706 10.1038/sj.onc.1203401

[bib2] Youle RJ, Strasser A. The BCL-2 protein family: opposing activities that mediate cell death. Nat Rev Mol Cell Biol 2008; 9: 47–59. 18097445 10.1038/nrm2308

[bib3] Adams JM, Cory S. The Bcl-2 apoptotic switch in cancer development and therapy. Oncogene 2007; 26: 1324–1337. 17322918 10.1038/sj.onc.1210220 PMC2930981

[bib4] Kelly PN, Strasser A. The role of Bcl-2 and its pro-survival relatives in tumourigenesis and cancer therapy. Cell Death Differ 2011; 18: 1414–1424. 21415859 10.1038/cdd.2011.17 PMC3149740

[bib5] Tagscherer KE, Fassl A, Campos B, Farhadi M, Kraemer A, Bock BC et al. Apoptosis-based treatment of glioblastomas with ABT-737, a novel small molecule inhibitor of Bcl-2 family proteins. Oncogene 2008; 27: 6646–6656. 18663354 10.1038/onc.2008.259

[bib6] van Delft MF, Wei AH, Mason KD, Vandenberg CJ, Chen L, Czabotar PE et al. The BH3 mimetic ABT-737 targets selective Bcl-2 proteins and efficiently induces apoptosis via Bak/Bax if Mcl-1 is neutralized. Cancer Cell 2006; 10: 389–399. 17097561 10.1016/j.ccr.2006.08.027 PMC2953559

[bib7] Tse C, Shoemaker AR, Adickes J, Anderson MG, Chen J, Jin S et al. ABT-263: a potent and orally bioavailable Bcl-2 family inhibitor. Cancer Res 2008; 68: 3421–3428. 18451170 10.1158/0008-5472.CAN-07-5836

[bib8] Huber S, Oelsner M, Decker T, zum Buschenfelde CM, Wagner M, Lutzny G et al. Sorafenib induces cell death in chronic lymphocytic leukemia by translational downregulation of Mcl-1. Leukemia 2011; 25: 838–847. 21293487 10.1038/leu.2011.2

[bib9] Trivigno D, Essmann F, Huber SM, Rudner J. Deubiquitinase USP9x confers radioresistance through stabilization of Mcl-1. Neoplasia 2012; 14: 893–904. 23097624 10.1593/neo.12598 PMC3479835

[bib10] Maurer U, Charvet C, Wagman AS, Dejardin E, Green DR. Glycogen synthase kinase-3 regulates mitochondrial outer membrane permeabilization and apoptosis by destabilization of MCL-1. Mol Cell 2006; 21: 749–760. 16543145 10.1016/j.molcel.2006.02.009

[bib11] Inuzuka H, Shaik S, Onoyama I, Gao D, Tseng A, Maser RS et al. SCF(FBW7) regulates cellular apoptosis by targeting MCL1 for ubiquitylation and destruction. Nature 2011; 471: 104–109. 21368833 10.1038/nature09732 PMC3076007

[bib12] Schwickart M, Huang X, Lill JR, Liu J, Ferrando R, French DM et al. Deubiquitinase USP9X stabilizes MCL1 and promotes tumour cell survival. Nature 2010; 463: 103–107. 20023629 10.1038/nature08646

[bib13] Wick W, Hartmann C, Engel C, Stoffels M, Felsberg J, Stockhammer F et al. NOA-04 randomized phase III trial of sequential radiochemotherapy of anaplastic glioma with procarbazine, lomustine, and vincristine or temozolomide. J Clin Oncol 2009; 27: 5874–5880. 19901110 10.1200/JCO.2009.23.6497

[bib14] Tagscherer KE, Fassl A, Sinkovic T, Combs SE, Roth W. p53-dependent regulation of Mcl-1 contributes to synergistic cell death by ionizing radiation and the Bcl-2/Bcl-XL inhibitor ABT-737. Apoptosis 2012; 17: 187–199. 22002102 10.1007/s10495-011-0664-3

[bib15] Zhang C, Cai TY, Zhu H, Yang LQ, Jiang H, Dong XW et al. Synergistic antitumor activity of gemcitabine and ABT-737 *in vitro* and *in vivo* through disrupting the interaction of USP9X and Mcl-1. Mol Cancer Ther 2011; 10: 1264–1275. 21566062 10.1158/1535-7163.MCT-10-1091

[bib16] Peddaboina C, Jupiter D, Fletcher S, Yap JL, Rai A, Tobin RP et al. The downregulation of Mcl-1 via USP9X inhibition sensitizes solid tumors to Bcl-xl inhibition. BMC Cancer 2012; 12: 541. 23171055 10.1186/1471-2407-12-541 PMC3543233

[bib17] Gratas C, Sery Q, Rabe M, Oliver L, Vallette FM. Bak and Mcl-1 are essential for temozolomide induced cell death in human glioma. Oncotarget 2014; 5: 2428–2435. 24811082 10.18632/oncotarget.1642 PMC4058016

[bib18] Murphy AC, Weyhenmeyer B, Noonan J, Kilbride SM, Schimansky S, Loh KP et al. Modulation of Mcl-1 sensitizes glioblastoma to TRAIL-induced apoptosis. Apoptosis 2014; 19: 629–642. 24213561 10.1007/s10495-013-0935-2 PMC3938842

[bib19] Allen JC, Talab F, Zuzel M, Lin K, Slupsky JR. c-Abl regulates Mcl-1 gene expression in chronic lymphocytic leukemia cells. Blood 2011; 117: 2414–2422. 21220745 10.1182/blood-2010-08-301176

[bib20] Ding Q, He X, Hsu JM, Xia W, Chen CT, Li LY et al. Degradation of Mcl-1 by beta-TrCP mediates glycogen synthase kinase 3-induced tumor suppression and chemosensitization. Mol Cell Biol 2007; 27: 4006–4017. 17387146 10.1128/MCB.00620-06 PMC1900029

[bib21] Wertz IE, Kusam S, Lam C, Okamoto T, Sandoval W, Anderson DJ et al. Sensitivity to antitubulin chemotherapeutics is regulated by MCL1 and FBW7. Nature 2011; 471: 110–114. 21368834 10.1038/nature09779

[bib22] Zhong Q, Gao W, Du F, Wang X. Mule/ARF-BP1, a BH3-only E3 ubiquitin ligase, catalyzes the polyubiquitination of Mcl-1 and regulates apoptosis. Cell 2005; 121: 1085–1095. 15989957 10.1016/j.cell.2005.06.009

[bib23] Wang B, Xie M, Li R, Owonikoko TK, Ramalingam SS, Khuri FR et al. Role of Ku70 in deubiquitination of Mcl-1 and suppression of apoptosis. Cell Death Differ 2014; 21: 1160–1169. 24769731 10.1038/cdd.2014.42 PMC4207484

[bib24] Cox JL, Wilder PJ, Gilmore JM, Wuebben EL, Washburn MP, Rizzino A. The SOX2-interactome in brain cancer cells identifies the requirement of MSI2 and USP9X for the growth of brain tumor cells. PloS One 2013; 8: e62857. 23667531 10.1371/journal.pone.0062857 PMC3647065

[bib25] Stegeman S, Jolly LA, Premarathne S, Gecz J, Richards LJ, Mackay-Sim A et al. Loss of Usp9x disrupts cortical architecture, hippocampal development and TGFbeta-mediated axonogenesis. PloS One 2013; 8: e68287. 23861879 10.1371/journal.pone.0068287 PMC3702552

[bib26] Homan CC, Kumar R, Nguyen LS, Haan E, Raymond FL, Abidi F et al. Mutations in USP9X are associated with X-linked intellectual disability and disrupt neuronal cell migration and growth. Am J Hum Genet 2014; 94: 470–478. 24607389 10.1016/j.ajhg.2014.02.004 PMC3951929

[bib27] Mouchantaf R, Azakir BA, McPherson PS, Millard SM, Wood SA, Angers A. The ubiquitin ligase itch is auto-ubiquitylated *in vivo* and *in vitro* but is protected from degradation by interacting with the deubiquitylating enzyme FAM/USP9X. J Biol Chem 2006; 281: 38738–38747. 17038327 10.1074/jbc.M605959200

[bib28] Taya S, Yamamoto T, Kanai-Azuma M, Wood SA, Kaibuchi K. The deubiquitinating enzyme Fam interacts with and stabilizes beta-catenin. Genes Cells 1999; 4: 757–767. 10620020 10.1046/j.1365-2443.1999.00297.x

[bib29] Watson RL, Spalding AC, Zielske SP, Morgan M, Kim AC, Bommer GT et al. GSK3beta and beta-catenin modulate radiation cytotoxicity in pancreatic cancer. Neoplasia 2010; 12: 357–365. 20454507 10.1593/neo.92112 PMC2864473

[bib30] Azakir BA, Angers A. Reciprocal regulation of the ubiquitin ligase Itch and the epidermal growth factor receptor signaling. Cell Signal 2009; 21: 1326–1336. 19341794 10.1016/j.cellsig.2009.03.020

[bib31] Fedrigo CA, Grivicich I, Schunemann DP, Chemale IM, dos Santos D, Jacovas T et al. Radioresistance of human glioma spheroids and expression of HSP70, p53 and EGFr. Radiat Oncol 2011; 6: 156. 22077956 10.1186/1748-717X-6-156 PMC3223500

[bib32] Zheng X, Zhai B, Koivunen P, Shin SJ, Lu G, Liu J et al. Prolyl hydroxylation by EglN2 destabilizes FOXO3a by blocking its interaction with the USP9x deubiquitinase. Genes Dev 2014; 28: 1429–1444. 24990963 10.1101/gad.242131.114 PMC4083087

[bib33] Krajewski S, Krajewska M, Ehrmann J, Sikorska M, Lach B, Chatten J et al. Immunohistochemical analysis of Bcl-2, Bcl-X, Mcl-1, and Bax in tumors of central and peripheral nervous system origin. Am J Pathol 1997; 150: 805–814. 9060818 PMC1857882

[bib34] Leverson JD, Zhang H, Chen J, Tahir SK, Phillips DC, Xue J et al. Potent and selective small-molecule MCL-1 inhibitors demonstrate on-target cancer cell killing activity as single agents and in combination with ABT-263 (navitoclax). Cell Death Dis 2015; 6: e1590. 25590800 10.1038/cddis.2014.561 PMC4669759

[bib35] Pareja F, Macleod D, Shu C, Crary JF, Canoll PD, Ross AH et al. PI3K and Bcl-2 inhibition primes glioblastoma cells to apoptosis through downregulation of Mcl-1 and Phospho-BAD. Mol Cancer Res 2014; 12: 987–1001. 24757258 10.1158/1541-7786.MCR-13-0650

[bib36] Levesley J, Steele L, Taylor C, Sinha P, Lawler SE. ABT-263 enhances sensitivity to metformin and 2-deoxyglucose in pediatric glioma by promoting apoptotic cell death. PloS One 2013; 8: e64051. 23691145 10.1371/journal.pone.0064051 PMC3656874

[bib37] Oltersdorf T, Elmore SW, Shoemaker AR, Armstrong RC, Augeri DJ, Belli BA et al. An inhibitor of Bcl-2 family proteins induces regression of solid tumours. Nature 2005; 435: 677–681. 15902208 10.1038/nature03579

[bib38] Certo M, Del Gaizo Moore V, Nishino M, Wei G, Korsmeyer S, Armstrong SA et al. Mitochondria primed by death signals determine cellular addiction to antiapoptotic BCL-2 family members. Cancer Cell 2006; 9: 351–365. 16697956 10.1016/j.ccr.2006.03.027

[bib39] Chen HC, Kanai M, Inoue-Yamauchi A, Tu HC, Huang Y, Ren D et al. An interconnected hierarchical model of cell death regulation by the BCL-2 family. Nat Cell Biol 2015; 17: 1270–1281. 26344567 10.1038/ncb3236 PMC4589531

[bib40] Miyashita T, Reed JC. Tumor suppressor p53 is a direct transcriptional activator of the human bax gene. Cell 1995; 80: 293–299. 7834749 10.1016/0092-8674(95)90412-3

[bib41] Oda E, Ohki R, Murasawa H, Nemoto J, Shibue T, Yamashita T et al. Noxa, a BH3-only member of the Bcl-2 family and candidate mediator of p53-induced apoptosis. Science 2000; 288: 1053–1058. 10807576 10.1126/science.288.5468.1053

[bib42] Lowe SW, Schmitt EM, Smith SW, Osborne BA, Jacks T. p53 is required for radiation-induced apoptosis in mouse thymocytes. Nature 1993; 362: 847–849. 8479522 10.1038/362847a0

[bib43] England B, Huang T, Karsy M. Current understanding of the role and targeting of tumor suppressor p53 in glioblastoma multiforme. Tumour Biol 2013; 34: 2063–2074. 23737287 10.1007/s13277-013-0871-3

[bib44] Ontikatze T, Rudner J, Handrick R, Belka C, Jendrossek V. Dihydroartemisinin is a hypoxia-active anti-cancer drug in colorectal carcinoma cells. Front Oncol 2014; 4: 116. 24904829 10.3389/fonc.2014.00116 PMC4032948

