# Supplementary figures and images for: Deubiquitylating enzyme USP9x regulates radiosensitivity in glioblastoma cells by Mcl-1-dependent and -independent mechanisms
Source: Cell Death Dis. 2016 Jan 14;7(1):e2039–. doi: 10.1038/cddis.2015.405 (PMC4816183; doi:10.1038/cddis.2015.405)

## Slide 1
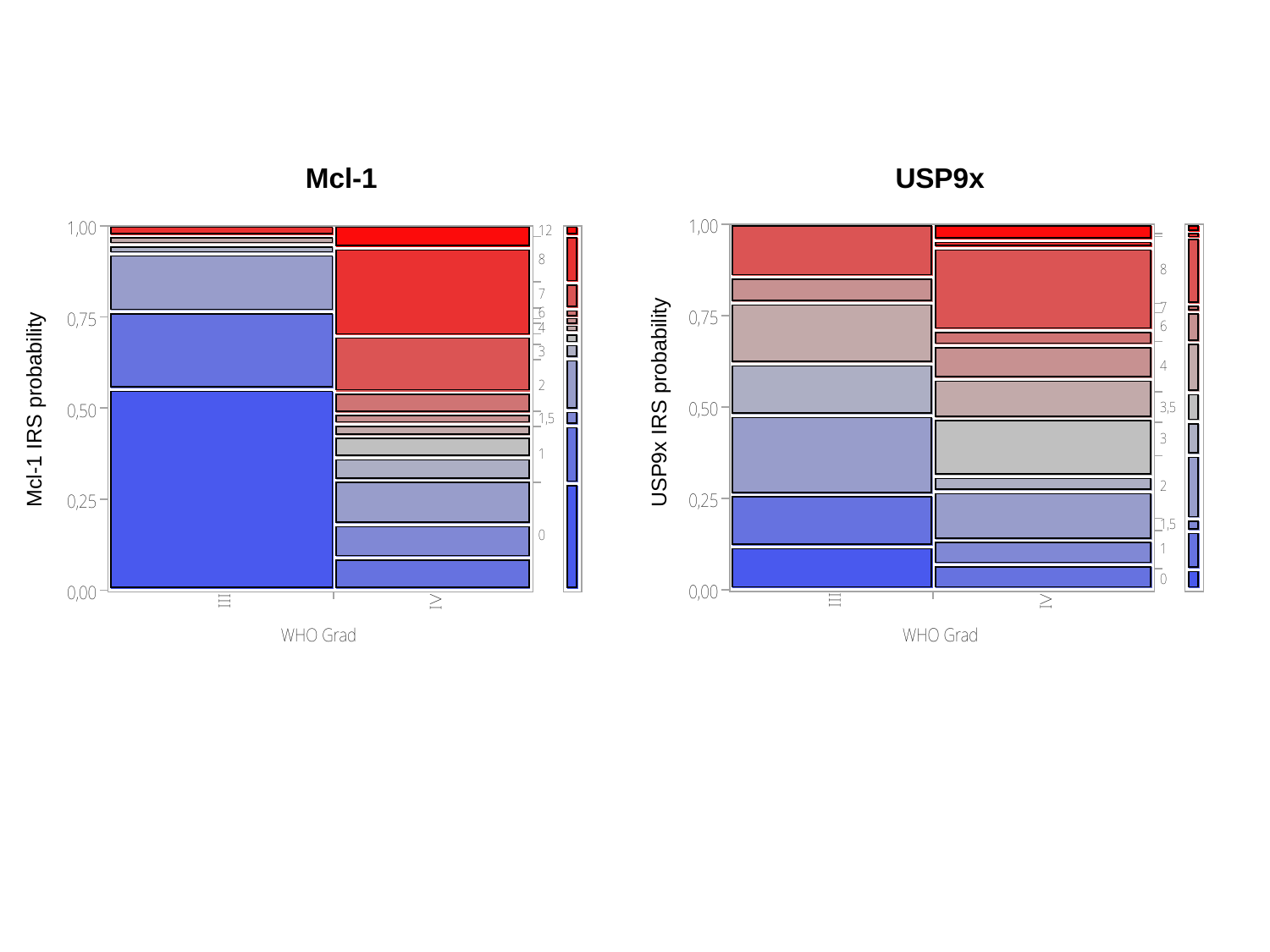

Mcl-1
USP9x
USP9x IRS probability
Mcl-1 IRS probability

Supplement: Supplementary Figure S1 [file cddis2015405x1.ppt]

## Slide 1
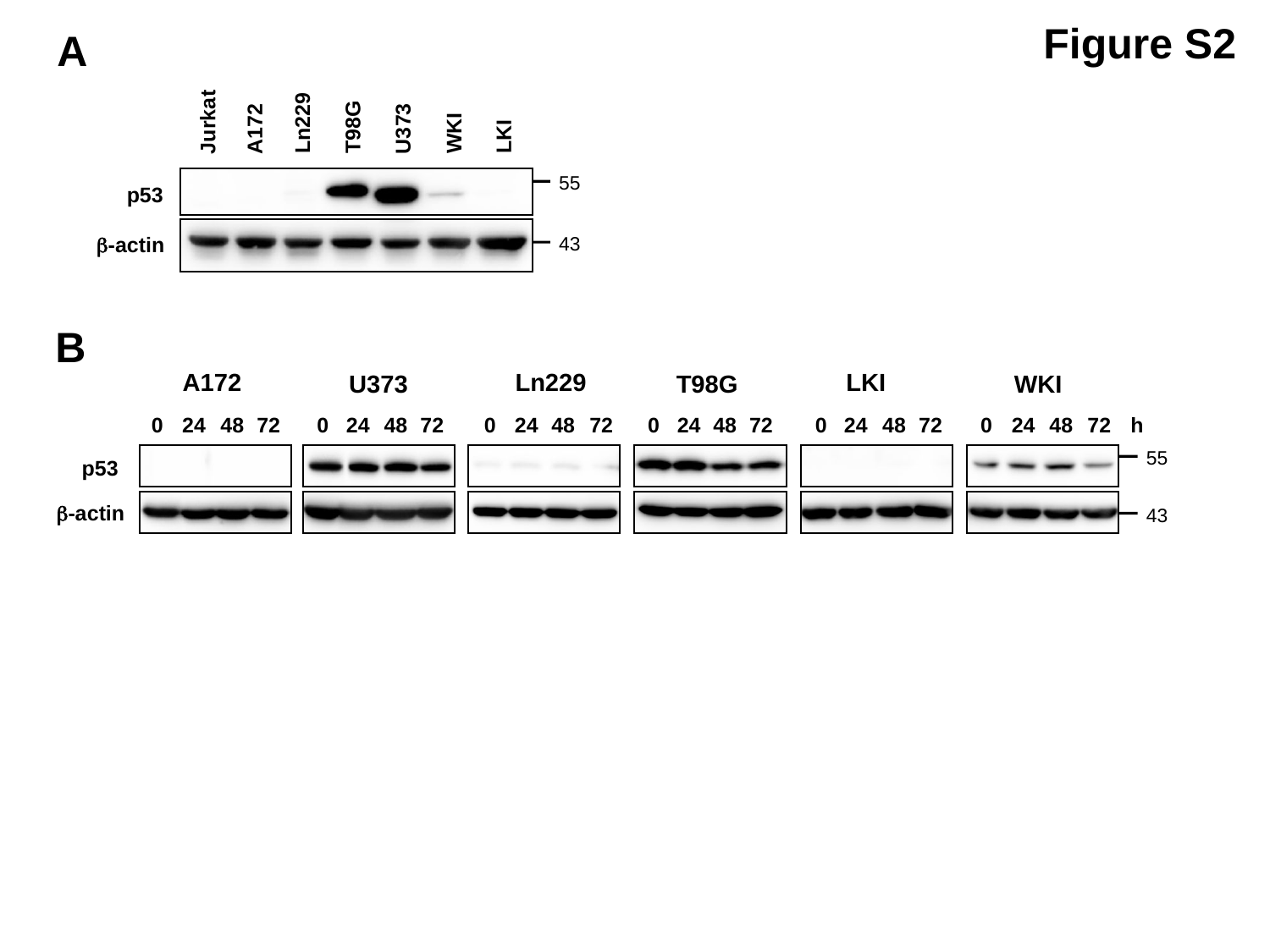

Figure S2
A
Jurkat
Ln229
T98G
A172
U373
WKI
LKI
55
p53
β-actin
43
B
A172
U373
0
24
48
72
0
24
48
72
Ln229
LKI
T98G
WKI
0
24
48
72
0
24
48
72
0
24
48
72
0
24
48
72
h
55
p53
β-actin
43

Supplement: Supplementary Figure S2 [file cddis2015405x2.ppt]

## Slide 1
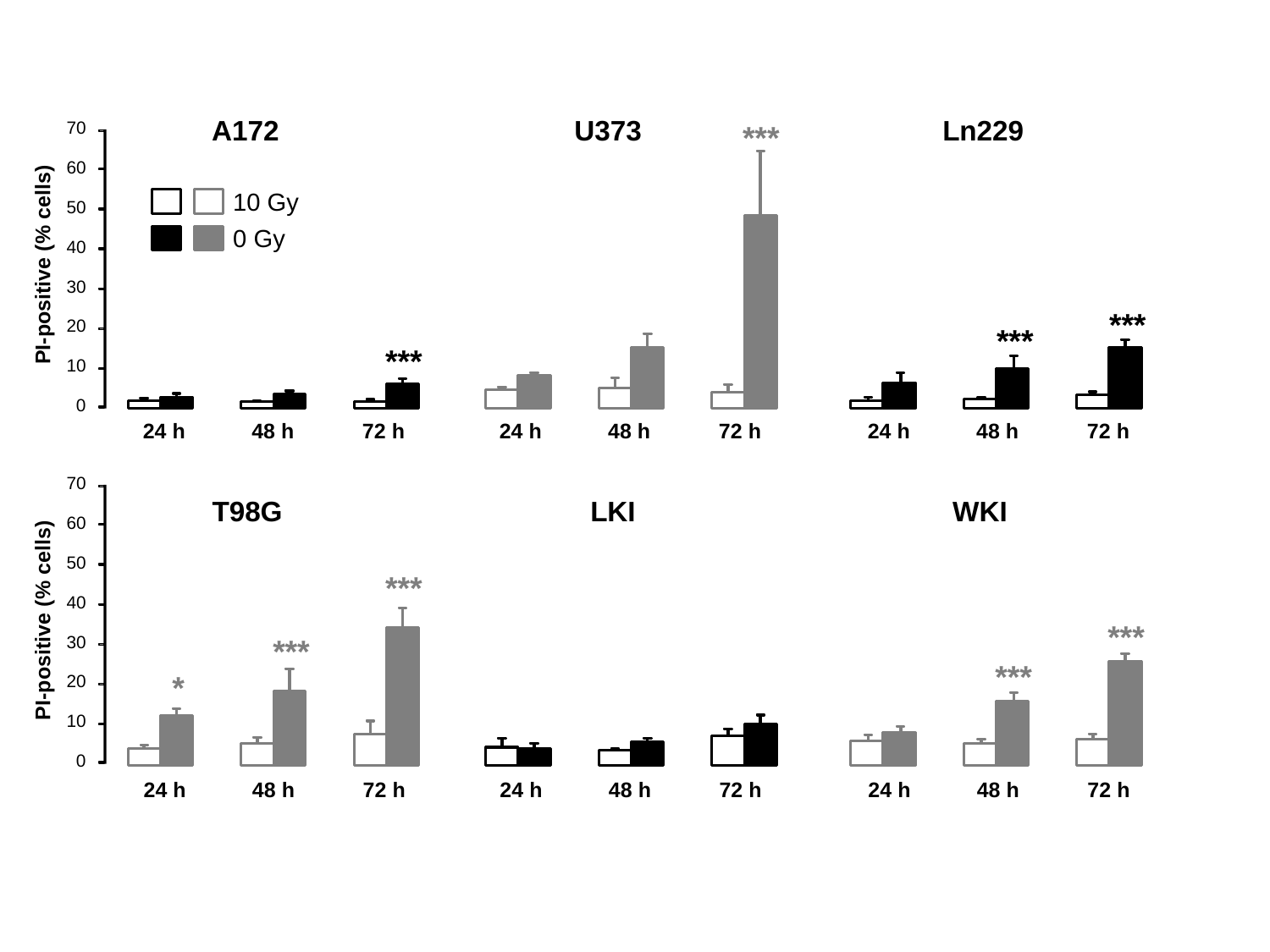

***
A172
U373
Ln229
70
60
50
40
PI-positive (% cells)
30
20
10
0
10 Gy
0 Gy
***
***
***
24 h
48 h
72 h
24 h
48 h
72 h
24 h
48 h
72 h
70
60
50
40
PI-positive (% cells)
30
20
10
0
T98G
LKI
WKI
***
***
***
***
*
24 h
48 h
72 h
24 h
48 h
72 h
24 h
48 h
72 h

Supplement: Supplementary Figure S3 [file cddis2015405x3.ppt]

## Slide 1
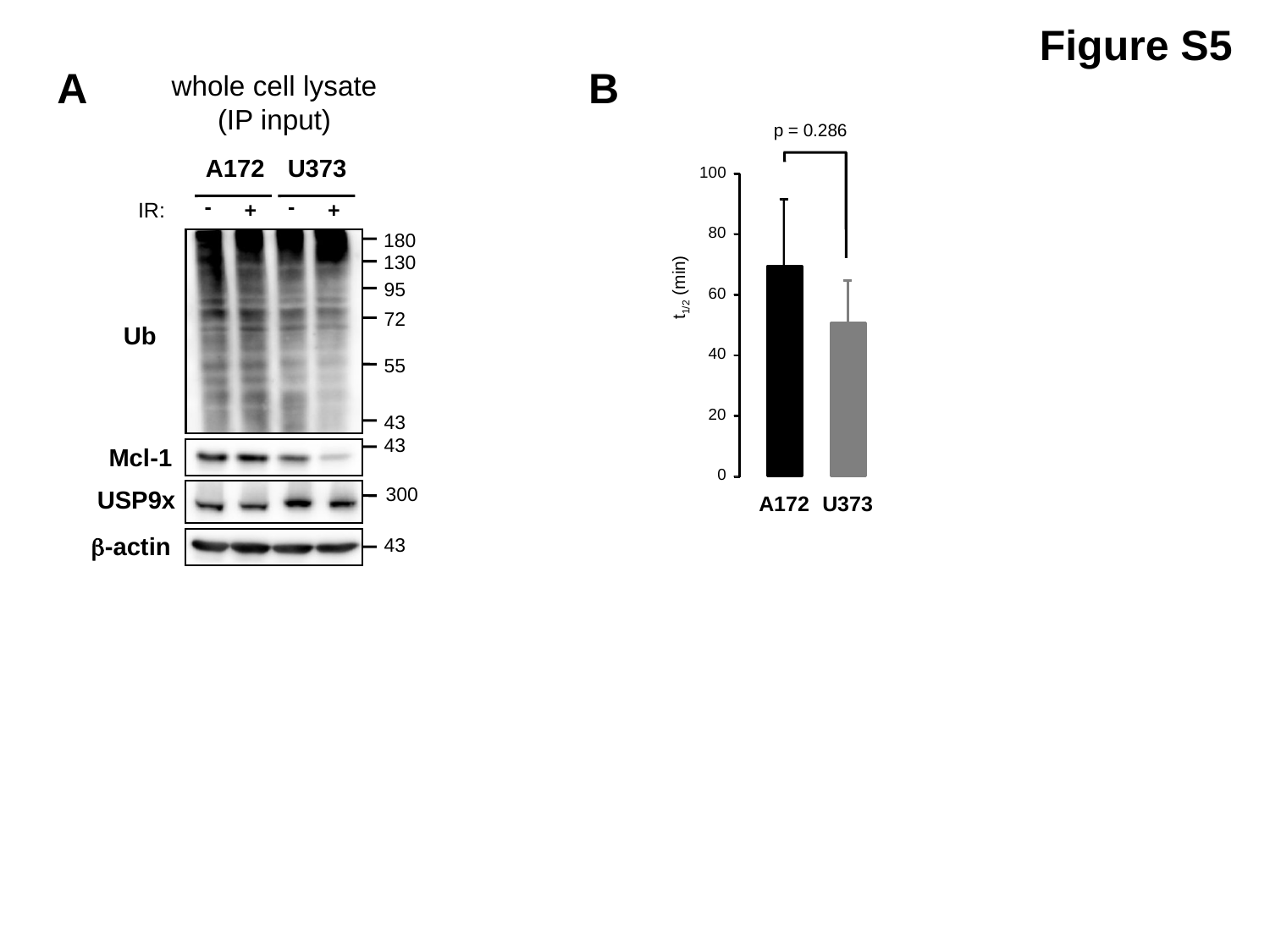

Figure S5
A
B
whole cell lysate
(IP input)
A172
U373
-
+
-
+
IR:
180
130
95
72
55
43
Ub
43
Mcl-1
300
USP9x
β-actin
43
p = 0.286
100
80
60
40
20
0
A172
U373
t1/2 (min)

Supplement: Supplementary Figure S5 [file cddis2015405x5.ppt]
