# Supplementary material for: Deubiquitylating enzyme USP9x regulates radiosensitivity in glioblastoma cells by Mcl-1-dependent and -independent mechanisms
Source: Cell Death Dis. 2016 Jan 14;7(1):e2039–. doi: 10.1038/cddis.2015.405 (PMC4816183; doi:10.1038/cddis.2015.405)
Supplement: Supplementary Figure S4 [file cddis2015405x4.ppt]

## Slide 1
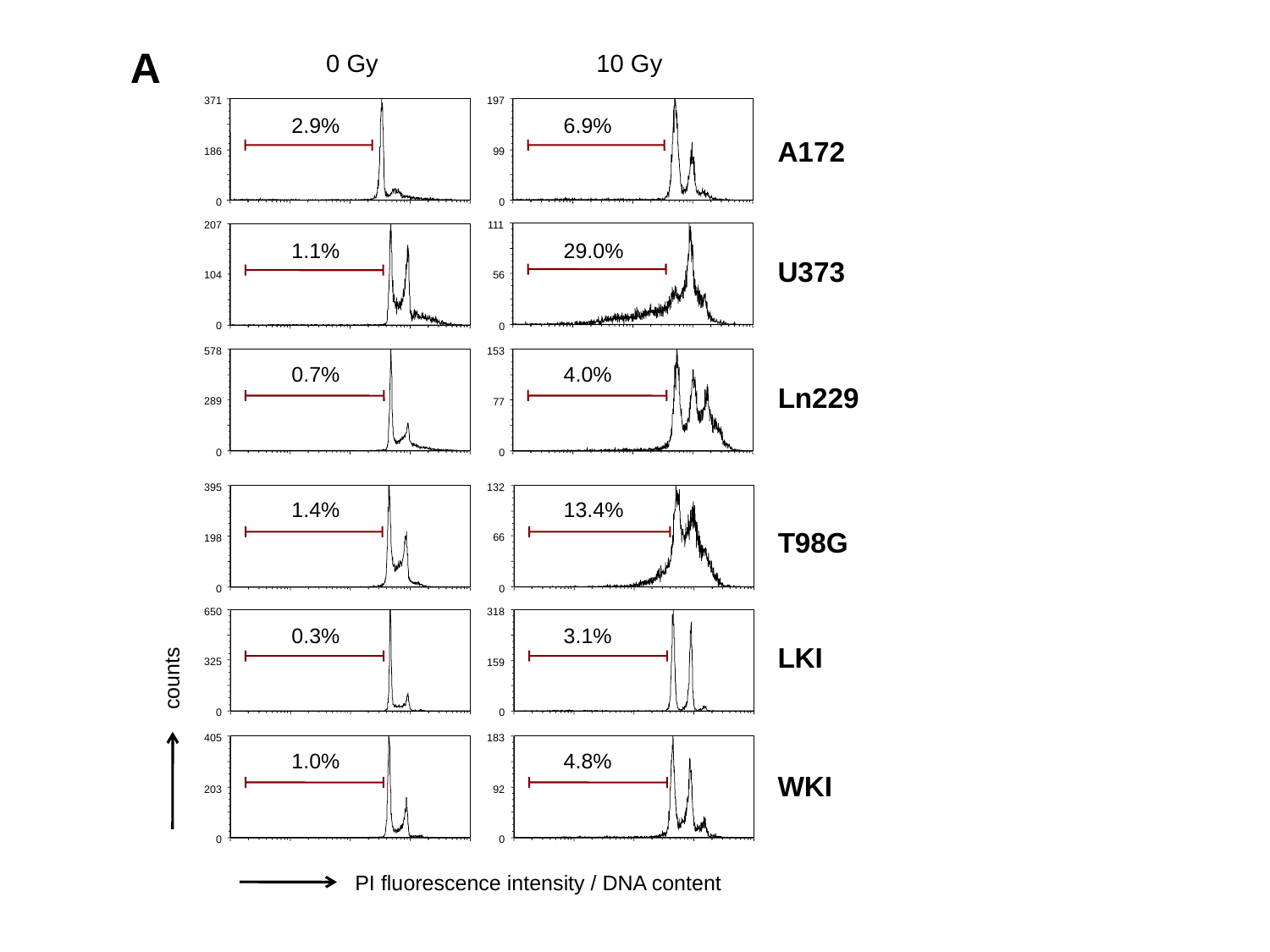

A
0 Gy
10 Gy
371
186
0
197
99
0
207
104
0
111
56
0
578
289
0
153
77
0
395
198
0
132
66
0
650
325
0
318
159
0
405
203
0
183
92
0
A172
U373
Ln229
T98G
LKI
counts
WKI
PI fluorescence intensity / DNA content
2.9%
6.9%
1.1%
29.0%
0.7%
4.0%
1.4%
13.4%
0.3%
3.1%
1.0%
4.8%

## Slide 2
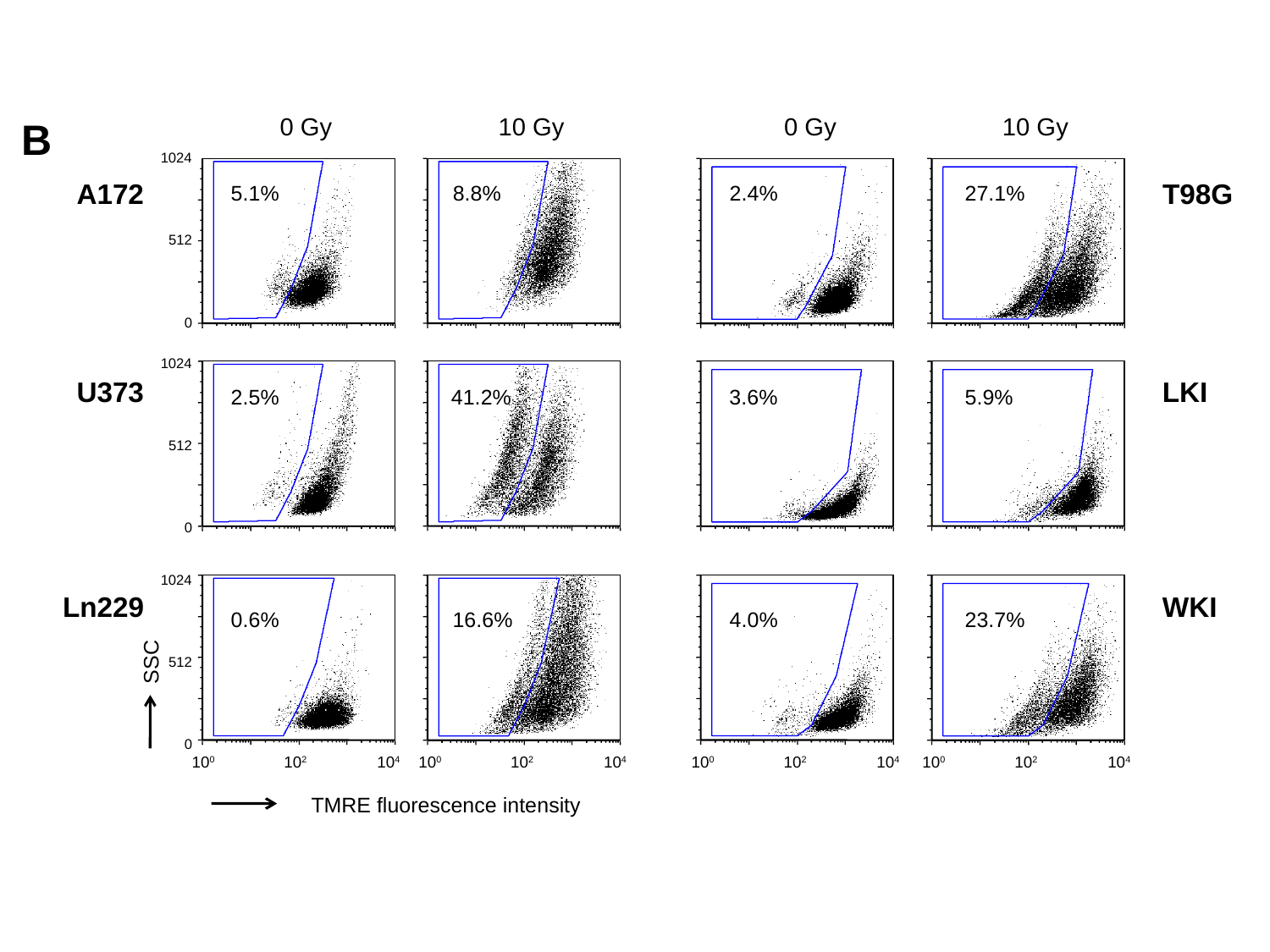

0 Gy
10 Gy
0 Gy
10 Gy
B
1024
512
0
A172
T98G
5.1%
8.8%
2.4%
27.1%
1024
512
0
U373
LKI
2.5%
41.2%
3.6%
5.9%
1024
512
0
Ln229
WKI
0.6%
16.6%
4.0%
23.7%
SSC
100
102
104
100
102
104
100
102
104
100
102
104
TMRE fluorescence intensity

## Slide 3
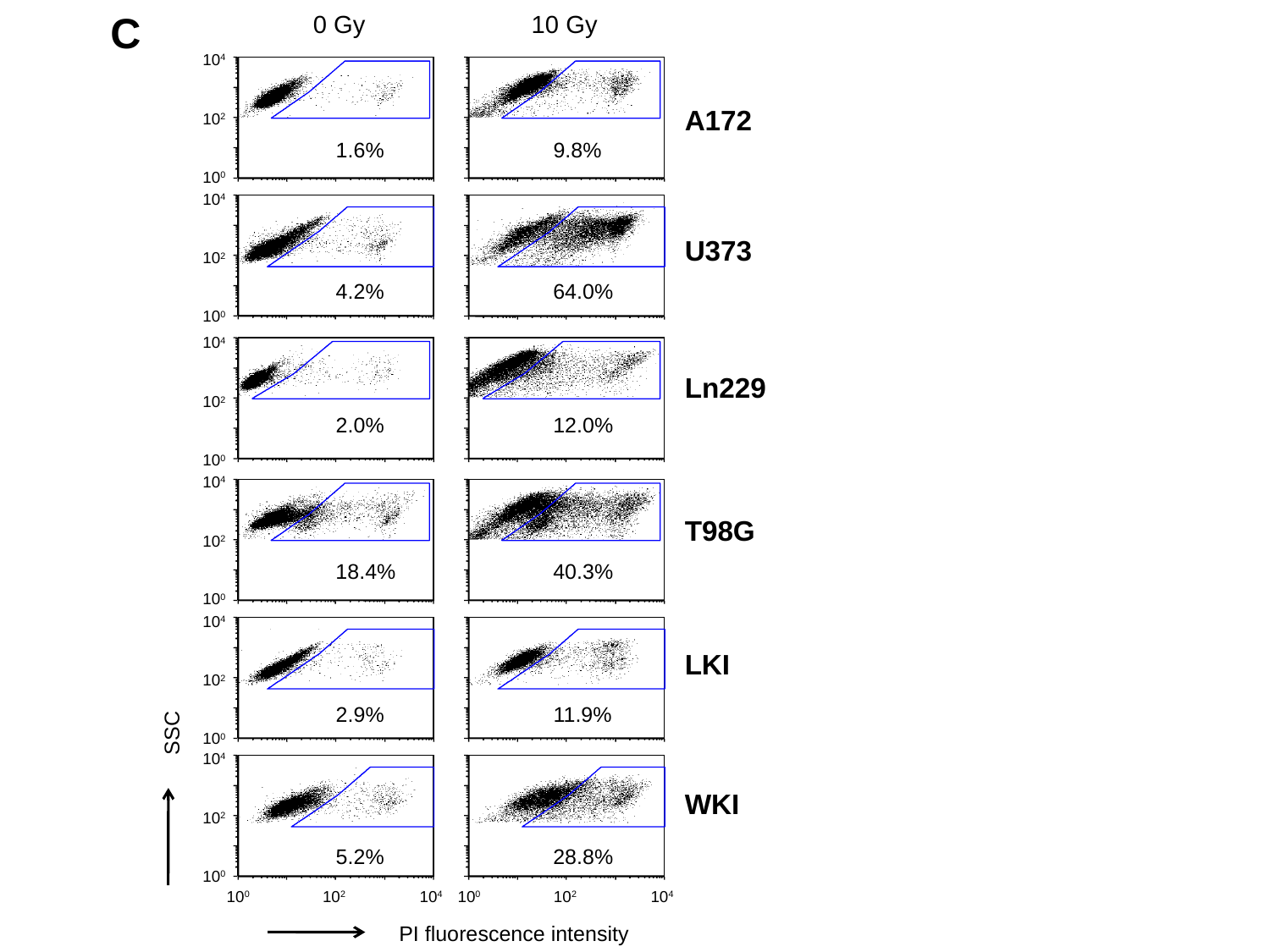

C
0 Gy
10 Gy
104
102
100
A172
1.6%
9.8%
104
102
100
U373
4.2%
64.0%
104
102
100
Ln229
2.0%
12.0%
104
102
100
T98G
18.4%
40.3%
104
102
100
LKI
2.9%
11.9%
SSC
104
102
100
4
WKI
5.2%
28.8%
100
102
104
100
102
104
PI fluorescence intensity
