# Supplementary material for: Deubiquitylating enzyme USP9x regulates radiosensitivity in glioblastoma cells by Mcl-1-dependent and -independent mechanisms
Source: Cell Death Dis. 2016 Jan 14;7(1):e2039–. doi: 10.1038/cddis.2015.405 (PMC4816183; doi:10.1038/cddis.2015.405)
Supplement: Supplementary Figure S7 [file cddis2015405x7.ppt]

## Slide 1
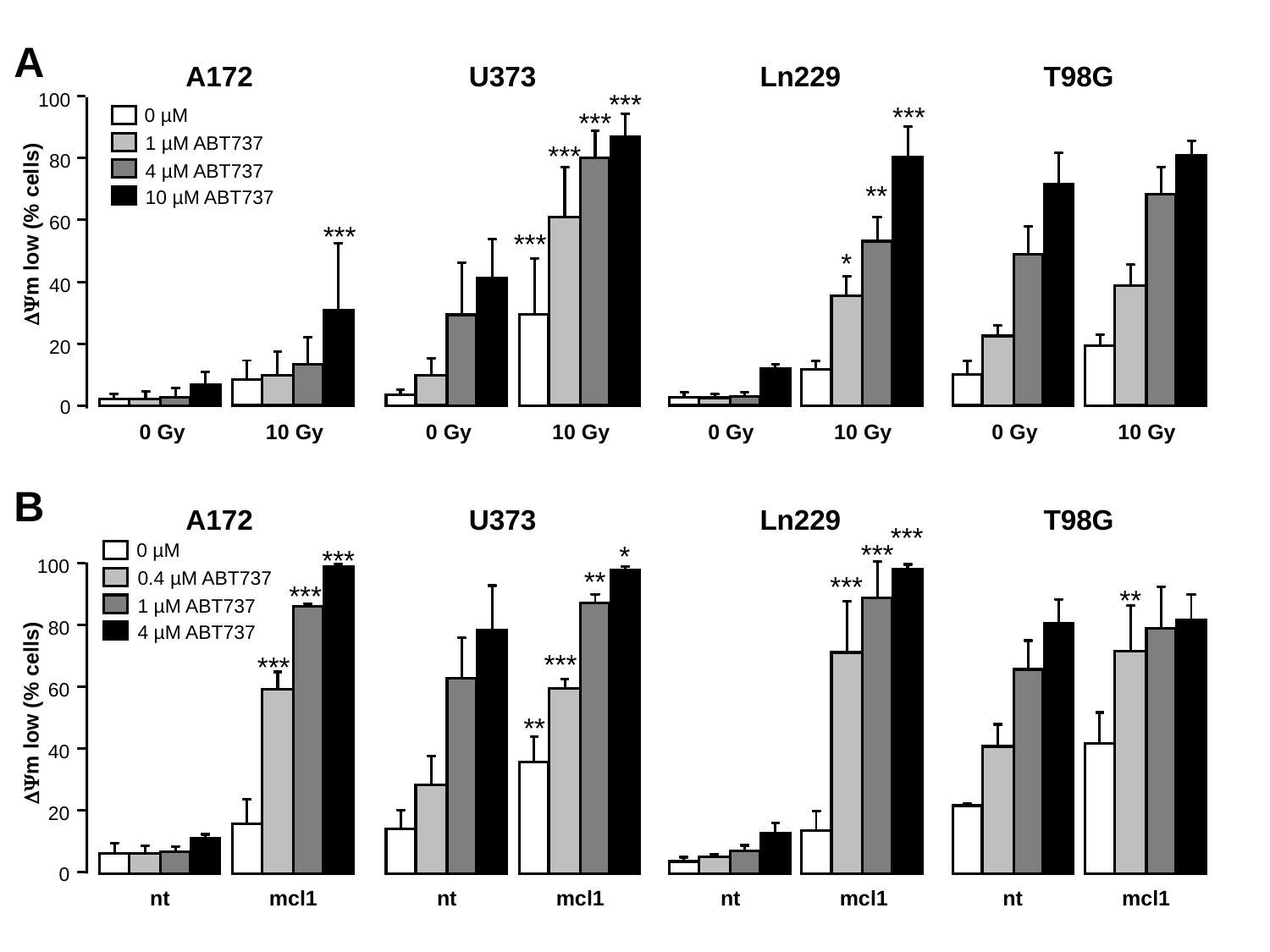

A
A172
U373
Ln229
T98G
***
100
80
60
40
20
0
***
***
0 µM
1 µM ABT737
4 µM ABT737
10 µM ABT737
***
**
***
m low (% cells)
***
*
0 Gy
10 Gy
0 Gy
10 Gy
0 Gy
10 Gy
0 Gy
10 Gy
B
A172
U373
Ln229
T98G
***
***
*
***
0 µM
0.4 µM ABT737
1 µM ABT737
4 µM ABT737
100
80
60
40
20
0
**
***
***
**
***
***
***
***
***
m low (% cells)
**
nt
mcl1
nt
mcl1
nt
mcl1
nt
mcl1
