# Supplementary material for: Deubiquitylating enzyme USP9x regulates radiosensitivity in glioblastoma cells by Mcl-1-dependent and -independent mechanisms
Source: Cell Death Dis. 2016 Jan 14;7(1):e2039–. doi: 10.1038/cddis.2015.405 (PMC4816183; doi:10.1038/cddis.2015.405)
Supplement: Supplementary Figure Legends [file cddis2015405x8.doc]

**Supplementary Figure S1:** Mcl-1 and USP9x expression was examined in astrocytoma (WHO grade III) and glioblastoma (WHO grade IV) tissue samples by immunohistochemical staining. After calculating the immunoreactive score (IRS) for Mcl-1 (right panel) and USP9x (left panel), a contingency analysis was performed. Results are shown in mosaic plots. Red color intensity indicates strong Mcl-1 or USP9x expression, while blue color intensity indicates weak Mcl-1 or USP9x expression. Mosaic plots show that Mcl-1 expression was upregulated more often than USP9x expression in tumor samples.

**Supplementary Figure S2:** (A) Cell lysates of established glioblastoma cell lines A172, T98G, U373, and Ln229 as well as primary glioblastoma cells WKI and LKI and Jurkat T lymphoma cells were separated by gel electrophoresis. (B) Glioblastoma cells were irradiated with 0 Gy10 Gy. 24 h, 48 h, and 72 h after irradiation, cells were lysed. p53 levels were analyzed by western blot. β-actin was used as loading control. High p53 levels were detected in non-irradiated T98G and U373 cells, whereas moderate p53 levels were detected in WKI cells. No increase in p53 level was observed 24-72 h after irradiation, suggesting an impaired p53 response in all glioblastoma cells in response to IR.

**Supplementary Figure S3:** A172, U373, Ln229, T98G, LKI, and WKI glioblastoma cells were irradiated with 0 Gy or 10 Gy. 24 h, 48 h, and 72 h after irradiation, cell death induction was analyzed by flow cytometry after staining with propidium iodide (PI, 10 µg/ml in PBS) for 30 min. PI-positive cells are considered dead. Most PI-positive cells were observed in U373, T98G, and WKI cells 72 h after irradiation. Flow cytometric data show mean values ± S.D. (T98G: n = 5; other cells: n = 4). Significance was calculated to the respective non-irradiated cells. *: p<0.05, **: p<0.01, ***: p<0.001.

**Supplementary Figure S4:** 72 h after irradiation with 0 Gy or 10 Gy, glioblastoma cells were analyzed by flow cytometry. (A) Representative histograms of DNA fragmentation. The marker indicates cells with fragmented DNA. (B) Representative dot plots of ΔΨm dissipation. The gate indicates cells with dissipated mitochondrial potential (ΔΨm low). (C) Representative dot plots analyzing cell death by propidium iodide (PI) exclusion dye. The gate indicates PI-positive cells. PI-positive cells are considered dead.

**Supplementary Figure S5:** (A) Whole cell lysates that were used for precipitation experiments described in Fig. 4B and 4C were analyzed by western blot. Ubiquitylated proteins, USP9x and Mcl-1 levels were analyzed 48 h after irradiation with 0 Gy (IR: -) or 10 Gy (IR: +). (B) A172 and U373 cells were treated with 5 µM cycloheximide as described in Fig. 4 D and E. Cells were lysed at respective time points, and Mcl-1 levels were analyzed by western blot followed by a densitometric quantification. Subsequently, Mcl-1 half-life time was calculated. Bar diagrams show the average half-life time of Mcl-1 in non-irradiated A172 and U373 cells ± S.D. (n = 3). Mcl-1 stability remained unchanged in A172 cells but was significantly decreased in U373 cells 48 h after irradiation. Mcl-1 half-life was slightly, but insignificantly, shorter in U373 than in A172 cells.

**Supplementary Figure S6:** A172, U373, Ln229, and T98G cells were transfected with 50 nM siRNA targeting Mcl-1 (mcl1, A), 100 nM siRNA targeting USP9x (usp9x, B), or with the respective amount of non- targeting (nt) siRNA. 48 h later, cells were irradiated with 0 Gy or 10 Gy. 48 h after irradiation, ΔΨm dissipation (ΔΨm low) was analyzed by flow cytometry. Mcl-1 knock-down significantly increased IR-induced ΔΨm dissipation in all cell lines. USP9x knock-down significantly increased IR-induced ΔΨm dissipation in U373 and Ln229 cells. Flow cytometric data show mean values ± S.D. (A172, U373: n = 3; A172, U373: n = 5). Significance was calculated to the respective cells transfected with non-targeting siRNA or, where indicated by a line, to non-irradiated control cells. *: p<0.05, **: p<0.01, ***: p<0.001.

**Supplementary Figure S7:** (A) A172, U373, Ln229, and T98G cells were irradiated (0 Gy, 10 Gy) and treated with the Bcl-2/Bcl-xL inhibitor ABT737 (0 µM/solvent control, 1 µM, 4 µM, or 10 µM) immediately after irradiation. 48 h later, ΔΨm dissipation (ΔΨm low) was analyzed by flow cytometry. Flow cytometric data show mean values ± S.D. (A172, U373: n = 6; Ln229, T98G: n = 3). (B) A172, U373, Ln229, and T98G cells were transfected with 50 nM siRNA targeting Mcl-1 (mcl1) or the respective non-targeting (nt) siRNA. 24 h after transfection, cell were treated with ABT737 (0 µM/solvent control, 0.4 µM, 1 µM, or 4 µM). 48 h later, ΔΨm dissipation (ΔΨm low) was analyzed by flow cytometry. Flow cytometric data show mean values ± S.D. (n = 3). Significance was calculated to the respective non-irradiated cells. *: p<0.05, **: p<0.01, ***: p<0.001. Irradiation and Mcl-1 knock-down facilitates ABT737-induced ΔΨm dissipation in glioblastoma cells.
